# Publishers' Response to Post‐Publication Concerns About Clinical Research in Women's Health

**DOI:** 10.1111/1471-0528.18100

**Published:** 2025-02-26

**Authors:** Siddharth Shivantha, Nicole Ling Shan Au, Lyle Gurrin, Jim Thornton, Jeremy Nielsen, Ben W. Mol

**Affiliations:** ^1^ Department of Obstetrics and Gynaecology Monash University Clayton Australia; ^2^ Centre for Epidemiology and Biostatistics, School of Population and Global Health The University of Melbourne Parkville Australia; ^3^ Division of Child Health, Obstetrics and Gynaecology of Nottingham University University of Nottingham Nottingham UK; ^4^ Department of Obstetrics and Gynaecology Amsterdam University Medical Centre Amsterdam the Netherlands

**Keywords:** post‐publication review, trustworthiness, untrustworthy data, Women's health

## Abstract

**Objective:**

Potentially untrustworthy medical research is often identified after publication. We evaluated the effectiveness and efficiency of post‐publication review of such studies in women's health.

**Design:**

Cohort study.

**Sample:**

Potentially untrustworthy papers published in women's health journals.

**Methods:**

We wrote to the editors and publishers about potentially untrustworthy papers in women's health and requested an investigation according to the procedure established by the Committee of Publication Ethics (COPE).

**Main Outcome Measure:**

Study characteristics, investigation outcome classed as retraction, expression of concern (EoC), correction or no wrongdoing found, and time to decision. We also report the case completion rate per journal and publisher.

**Results:**

Between 7th November 2017 and 30th April 2024, we wrote to editors and publishers of 891 potentially untrustworthy papers published in 206 different journals. At present, 263 (30%) of 891 papers received an outcome, with 227 (86%) labelled as problematic [152 (58%) retracted; 75 (29%) EoC]. For articles with a decision, it took a median time of 38 months for editors and publishers to decide, with 13% of the flagged cases reaching a decision within 12 months.

**Conclusions:**

The current PPR process is inefficient and ineffective in assessing and removing untrustworthy data from the medical literature.

## 1 Introduction

Clinical research is the cornerstone of evidence‐based medicine and the foundation of modern healthcare. While the design and quality of studies can be discussed, it has, until now, been taken at face value that studies are at least based on reliable data. In recent years, it has become apparent that this assumption is incorrect [1, 2, 3, 4]. This is worrisome since these studies, specifically randomised clinical trials (RCTs), continue to inform medical guidelines and clinical practice.

Carlisle reported that among > 500 RCTs submitted to the journal Anaesthesia, 73 (14%) contained false data and 43 (8%) were deemed fatally flawed ‘zombie trials’ [1]. Based on this, Ioannidis estimated that there could be hundreds of thousands of problematic RCTs across all disciplines that go unchallenged and can even be harmful [3].

Alfirevic et al. using the Cochrane Pregnancy and Childbirth Group's trustworthiness screening tool (CPC‐TST) to assess 374 RCTs included in 18 Cochrane reviews, concluded that 95 RCTs (25%) merited exclusion for this reason, resulting in changes in 14 (80%) of the reviews, and important differences in the conclusions and/or implications in six (33%) [5]. A recent update of the International Evidence‐based Guideline for Polycystic Ovary Syndrome (PCOS) excluded 45% of eligible RCTs for not meeting trustworthiness criteria [6]. The estimate that at least 25% of RCTs used to inform clinical practice are untrustworthy seems justified [1, 3, 7].

Pre‐publication prevention is ideal, but a different approach is needed for already published studies. The Committee on Publication of Ethics (COPE), a charitable organisation that advocates for research integrity and ethical conduct, has established guidelines for post‐publication review and COPE member journals are bound by these [8]. If data integrity breaches are flagged, journals and publishers must investigate and, in case of wrongdoing, issue a formal public notice [9]. This serves as a critical juncture for investigating, correcting and, if needed, retracting potentially problematic data from the published literature.

We studied the effectiveness and efficiency of this process by assessing the response rate of editors and publishers of women's health journals to concerns raised about papers with potentially problematic data.

## 2 Method

### 2.1 Study Design

Between 7th November 2017 and 30th April 2024, we collated potentially untrustworthy clinical research papers, mainly on women's health. The papers were initially identified in the process of establishing systematic reviews and clinical guidelines. We also systematically assessed the work of authors in which problems had been identified, and we followed newly published RCTs. We used online databases, including PubMed, PubPeer, Google Scholar and the Retraction Watch database, to track author's previous work [10, 11]. Each identified RCT was cross‐checked against items in the checklist to assess Trustworthiness in Randomised Clinical Trials (TRACT) [12]. This checklist assesses trustworthiness based on seven domains (governance, author group, plausibility of intervention usage, timeframe, drop‐out rate, baseline characteristics and outcomes) [12]. Each domain has signalling questions answered as either no concerns, some concerns/no information, or major concerns [12]. Since the TRACT checklist was developed over the trajectory of this study, early assessments were less formal. Observational studies were also assessed using applicable checklist items. If concerns were raised in several domains, or if an article showed a single finding highly suggestive of data integrity breach, the article was flagged to the editors and publishers.

We have previously published examples of problematic papers [13, 14]. Our concerns were classified into eight categories: double publication, ethics concerns, fabricated data, plagiarism, similar outcome group characteristics, statistical discrepancies, study protocol deviations and trial registry discrepancies.

We informed journal editors and publishers via e‐mail, highlighting the concerns in detail and requested that the papers be assessed according to COPE guidelines [8]. We requested confirmation of receipt and resent if no response was received or we had no updates after 6 months.

### 2.2 Data Collection

All data were collected in a Google spreadsheet accessible to team members. We tabulated author(s), publication year, journal, publisher, institute, type of concern raised, type of study (RCT/observational), article link, initial e‐mail date sent to editors and publishers, and date of confirmation of receipt. Progress was denoted as retraction, Expression of Concern (EoC), correction, investigation concluded no action required or pending investigation. Initially, the last author (BWM) registered all findings; from 2023, the first author (SS) systematically verified all original e‐mails sent to editors and publishers, including the response date and outcome.

### 2.3 Statistical Analysis

We tabulated the type of outcomes received for each paper as retraction, EoC, correction or investigation concluded no action required and pending investigation. The median response time to issue an outcome was estimated using Kaplan–Meier curves. We note that there are several instances where the Kaplan–Meier curve provides an estimate of the median time to outcome despite < 50% of the papers in the sample being issued with an outcome. This is a consequence of including information from papers from which an outcome had not yet been issued. We performed subgroup analysis by publishers, journals and countries. Journals were categorised into five quartile rankings Q1, Q2, Q3, Q4 and unclassified according to the Scimago Journal and Country Ranking database [15]. We also calculated the time to response between the initial date of correspondence with editors and/or publishers and the final decision date. For each journal and publisher, we calculated the median time to response for those papers using Kaplan–Meier curves.

All statistical analysis was conducted using R statistical software (version 4.4.0). The analysis included outcomes as of 30th April 2024.

## 3 Results

**FIGURE 1:**
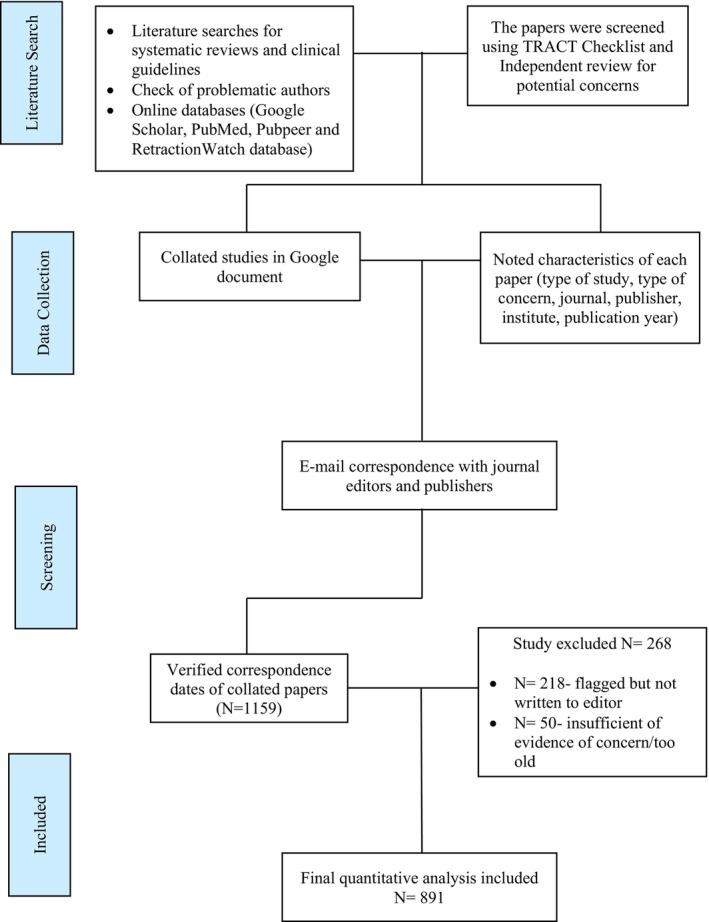
Flowchart of the number of papers analysed.

Our initial database contained 1159 potentially untrustworthy papers (Figure 1). We excluded 268 papers where we had not yet written to editors (*N* = 218), or we considered the paper to be too old/insufficient evidence for concern at this point (*N* = 50).

### 3.1 Baseline Characteristics

Table 1 presents the publication year, type of study and region of the studied papers. Most were published after 2010. More than half were RCTs.

**TABLE 1:** Baseline characteristics of papers.

	Total *N* = 891	Investigation concluded				Pending investigation *N* = 628
		Retraction *N* = 152	Expression of concern *N* = 75	Correction *N* = 6	Investigation concluded no action *N* = 30	
**Publication year**						
< 2000	12	1 (8%)	3 (25%)	0 (0%)	2 (17%)	6 (50%)
2000–2010	145	27 (19%)	19 (13%)	0 (0%)	5 (3%)	94 (65%)
2010–2020	527	89 (17%)	47 (9%)	4 (1%)	11 (2%)	376 (71%)
2020‐Present	207	35 (17%)	6 (3%)	2 (1%)	12 (6%)	152 (73%)
**Type of study**						
Observational	362	40 (11%)	25 (7%)	1 (0.4%)	5 (1%)	291 (80%)
RCT	529	112 (21%)	50 (9.5%)	5 (0.9%)	25 (4.7%)	337 (64%)
**Country of origin**						
Middle East	789	141 (18%)	72 (9%)	6 (1%)	19 (2%)	551 (70%)
Europe	55	6 (11%)	2 (4%)	0 (0%)	3 (5%)	44 (80%)
Asia	40	4 (10%)	1 (2%)	0 (0%)	8 (20%)	27 (68%)
Other (USA, Brazil and Tunisia)	7	0 (0%)	0 (0%)	0 (0%)	0 (0%)	7 (100%)
**Year 1st email sent**						
2017	2	2 (100%)	0 (0%)	0 (0%)	0 (0%)	0 (0%)
2019	19	5 (26%)	12 (64%)	0 (0%)	1 (5%)	1 (5%)
2020	62	34 (54%)	10 (16%)	0 (0%)	2 (4%)	16 (26%)
2021	187	64 (34%)	18 (10%)	2 (1%)	7 (4%)	96 (51%)
2022	351	39 (11%)	27 (8%)	4 (1%)	8 (2%)	273 (78%)
2023	199	8 (4%)	8 (4%)	0 (0%)	12 (6%)	171 (86%)
2024 (until April '24)	71	0 (0%)	0 (0%)	0 (0%)	0 (0%)	71 (100%)

*Note*: % Calculated from the total number of cases.

The number of cases flagged increased over time. The majority of cases were authored from countries in the Middle East (Egypt = 674, Iran = 95, Saudi Arabia = 10, and Turkey = 8), followed by Europe (Italy = 43, Germany = 9) and Asia (India = 26, China = 10).

### 3.2 Outcome of Editorial Investigations

As of 30th April 2024, 263/891 papers (29%) had received a final decision: retraction (152/263, 58%) and EoC (75/263, 29%) made up 86% of the decisions. There were 30 papers (11%) where, according to the investigation, no editorial action was required, while six papers had an erratum and subsequent corrections. The remaining 628 papers (71%) were yet to receive a decision.

### 3.3 Summary of Concern of Retracted and Expression of Concern Papers

Among the 227 papers that were retracted or received an EoC, 134 papers (69%) received said outcomes due to the presence of false data (retracted due to false data, *N* = 87; EoC due to false data, *N* = 47). Moreover, 43/227 (19%) papers received a retraction (*N* = 26) or an EoC (*N* = 17) due to plagiarism. One paper was retracted because of a lack of ethics approval and another because of the double publication.

### 3.4 Outcome Response Time

**FIGURE 2:**
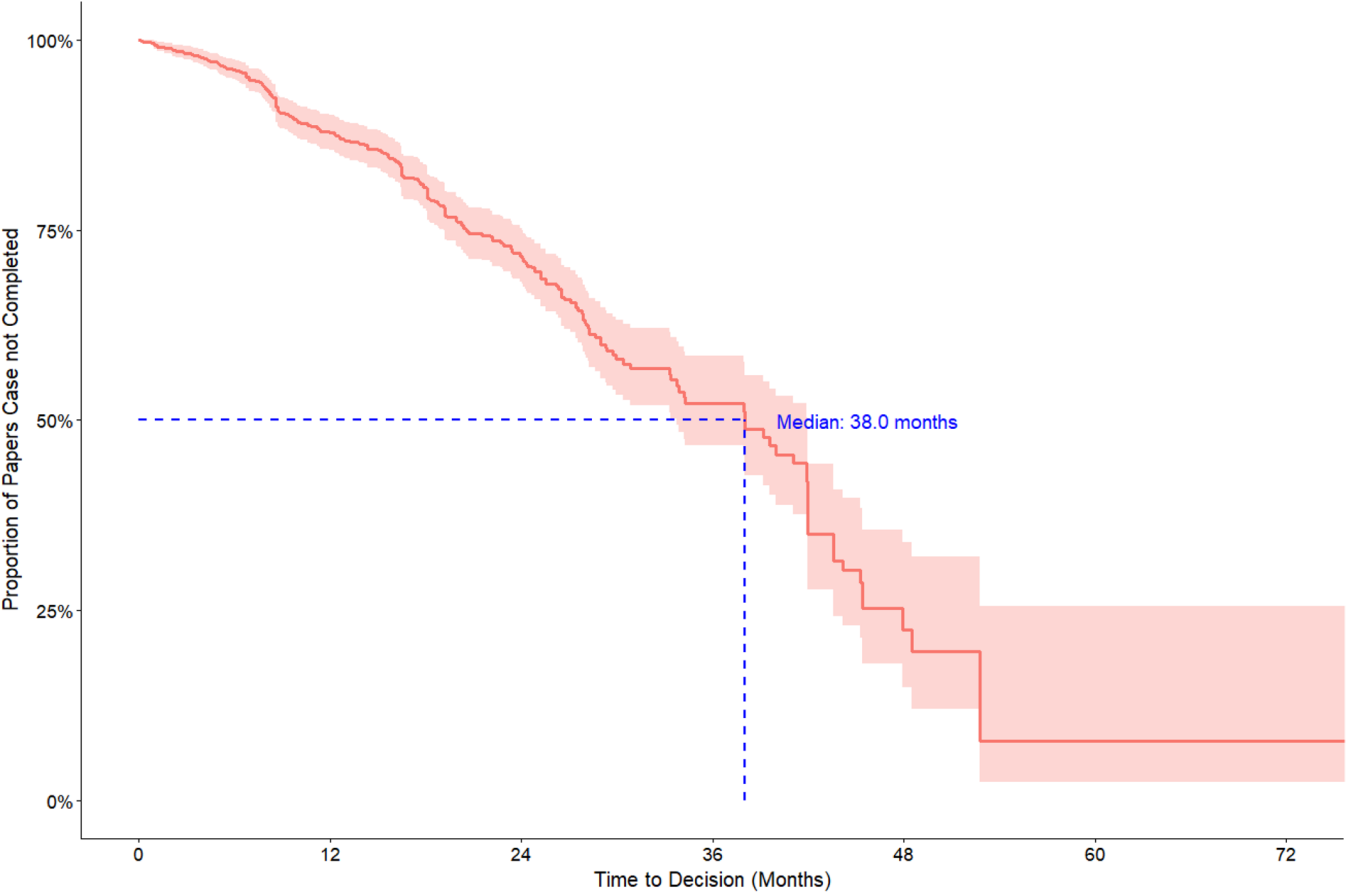
Kaplan‐Meier curve of time to case completion (all journals and publishers) up to 60 months, with right censoring for cases still“pending investigation” at the study end date.

The median time editors and publishers took to issue any outcome (retraction, EoC, correction, or investigation concluded no action) was 38 months, with only 13% of papers receiving conclusive outcomes within the first 12 months (Figure 2).

### 3.5 Outcomes by Journal

We wrote to 206 journals published by 80 publishers or societies. The full case completion rates by journal, discipline and journal base region are provided in Table S1. The flagged papers spanned 31 disciplines, predominantly women's health‐related studies.

Of 210 journals, 54 issued responses, while the remaining 156 journals (each with between 1 and 49 concerns, mostly flagged due to the presence of potential false data) had not finalised any conclusions. Among the Q1 journals, Fertility and Sterility (57 cases) resolved 39% (22/57) of cases, with a median time to conclusion of 31 months (Table 2). Moreover, 76% of resolved cases by Fertility and Sterility were retractions (*N* = 14) or EoC (*N* = 2), the highest of all Q1 journals. The International Journal of Gynaecology and Obstetrics had the most papers flagged from all Q1 journals (*N* = 67), with a case completion rate of 33% and 57% of the completed cases resulting in a retraction (median time to response of 34 months) The European Journal of Contraception and Reproductive Health Care had the highest case completion rate (19/24, 75%), with 18 out of 19 completed cases receiving retractions after a median time to decision of 22 months [16, 17].

**TABLE 2:** Journal case completion rate.

Journal *N* = 206	Q ranking	Publisher	Number of flagged papers *N* = 891	Case completion rate (%)	Retraction *N* = 152	Expression of concern *N* = 75	Correction *N* = 6	Investigation concluded no action *N* = 30	Pending investigation *N* = 628	Median time to response (Months) *assuming 30 days per month
Journal of Maternal‐Fetal and Neonatal Medicine	Q2	Taylor & Francis	78 (8.8%)	29 (37%)	11 (38%)	18 (62%)	0 (0%)	0 (0%)	49 (63%)	28
International Journal of Gynecology and Obstetrics	Q1	Wiley Blackwell	67 (7.5%)	22 (33%)	13 (57%)	0 (0%)	0 (0%)	10 (43%)	44 (66%)	34
Archives of Gynecology and Obstetrics	Q2	Springer	60 (6.7%)	31 (52%)	15 (48%)	16 (52%)	0 (0%)	0 (0%)	29 (48%)	38
Fertility Sterility	Q1	Elsevier	57 (6.4%)	22 (37%)	14 (64%)	2 (9%)	1 (5%)	4 (18%)	36 (63%)	31
European Journal of Obstetrics and Gynecology and Reproductive Biology	Q2	Elsevier	38 (4.3%)	16 (42%)	14 (88%)	2 (12%)	0 (0%)	0 (0%)	22 (58%)	28
Gynecological Endocrinology	Q2	Taylor & Francis	27 (3.0%)	7 (26%)	5 (71%)	2 (29%)	0 (0%)	0 (0%)	20 (74%)	45
European Journal of Contraception & Reproductive Health Care	Q2	Taylor & Francis	24 (2.7%)	19 (79%)	18 (95%)	0 (0%)	1 (5%)	0 (0%)	6 (25%)	22
Journal of Obstetrics and Gynaecology	Q3	Taylor & Francis	23 (2.6%)	12 (52%)	12 (100%)	0 (0%)	0 (0%)	0 (0%)	11 (48%)	28
Journal of Obstetrics and Gynaecology Research	Q2	Wiley Blackwell	21 (2.4%)	5 (24%)	2 (50%)	1 (25%)	1 (25%)	0 (0%)	17 (81%)	42
Reproductive Biomedicine Online	Q1	Elsevier	20 (2.2%)	5 (25%)	5 (100%)	0 (0%)	0 (0%)	0 (0%)	15 (75%)	44

*Note*: % Calculated from the number of completed cases.

For European‐based journals, the case completion rate was 34%, with 59% of completed cases ending in retraction and 28% in an EoC. For US‐based journals, the case completion rate was 32%, with 52% of completed cases ending in retraction and 32% in an EoC. None of the Middle East‐based journals reached a decision on the 46 cases flagged.

### 3.6 Outcomes by Publisher

The four publishers with the most cases raised were Elsevier, Taylor & Francis, Springer, and Wiley‐Blackwell (Table 3). Case completion rates varied between 30% and 42%. Combined retraction and EoC rates for decisions taken were high, varying between (70/71, 99%) for Taylor & Francis and (68%) for Wiley‐Blackwell. Full case completion rates of all publishers are listed in Table S2. Karger had the highest case completion rate (7/9, 78%), with a median time of 7 months to resolve a case. Publisher Termedia did not resolve any of the 11 flagged cases.

**TABLE 3:** Publisher case completion rate.

Status	Informed papers *N* = 891	Case completion (%)	Retraction *N* = 152	Expression of concern *N* = 75	Correction *N* = 6	Investigation concluded no action *N* = 30	Pending investigation *N* = 628	Median time to response (Months), *assuming 30 days per month
Elsevier	204 (23%)	61 (30%)	43 (70%)	10 (16%)	2 (3%)	6 (10%)	143 (70%)	42
Taylor & Francis	170 (19%)	70 (41%)	50 (71%)	20 (28%)	0 (0%)	1 (2%)	100 (58%)	28
Springer	154 (17%)	50 (33%)	26 (52%)	18 (36%)	2 (4%)	4 (8%)	103 (67%)	25
Wiley Blackwell	125 (14%)	37 (30%)	17 (46%)	8 (22%)	1 (3%)	11 (30%)	88 (70%)	34
Wolters Kluwer	45 (5.1%)	22 (49%)	5 (23%)	14 (63%)	0 (0%)	3 (14%)	23 (51%)	18
Oxford University Press	13 (1.5%)	3 (23%)	1 (33%)	0 (0%)	0 (0%)	2 (67%)	10 (77%)	NA
Termedia	11 (1.2%)	0 (0%)	0 (0%)	0 (0%)	0 (0%)	0 (0%)	11 (100%)	NA
Karger	9 (1.0%)	7 (78%)	2 (29%)	4 (57%)	0 (0%)	1 (14%)	2 (22%)	7

*Note*: The percentage in the “Informed papers” column is the percentage of the column total. The percentage in the “Case completion” and “Pending investigation” columns are the percentages that case completion and pending investigation represent of the “Informed papers” – these two percentages add up to 100%. The percentages in the remaining four columns (“Retraction”, “Expression of concern”, “Correction” and “Investigation concluded no action”) are percentages of the number of case completions and add up to 100%.

## 4 Discussion

### 4.1 Main Findings

In this series of almost 900 papers, we found delays in academic publishers' responses to flagged problematic papers. A decision was reached on less than a third of flagged cases over a median time of over 3 years, and only 13% was resolved within a year. About 85% of the decisions were retractions or EoC.

### 4.2 Strengths and Limitations

Our survey is large and up to date. The high rate of retractions and EoC among resolved cases suggests that our methods for identifying problematic papers are robust. Although we flagged > 900 potentially problematic papers, mostly in women's health, this probably represents only a small fraction of the problematic papers circulating in the literature if we assume a rate of 5%–10% of all research papers and 25% of RCTs [3]. Our estimates do not apply to predatory journals where it is often difficult to contact editors and publishers [18]. We did not assess papers published in known predatory journals.

### 4.3 Comparison With Previous Research

Our results echo previous studies' concerns about the extent of untrustworthy data in women's health and other disciplines [1]. Chambers et al. reported that article retractions within obstetrics and gynaecology were increasing, with the most frequently cited reasons being plagiarism, fabricated results, article duplication and compromised peer review [2]. Moylan and Kowalczuk studied retraction messages and concluded that checklists and templates for various classes of retraction notices would increase transparency in retraction messages [19]. A recent Nature article reported that the retraction rate for European‐based biomedical papers has quadrupled between 2000 and 2021, with the majority stemming from data and image manipulation [20]. This is partly due to whistle‐blowers flagging untrustworthy papers and the increased use of digital tools such as plagiarism detectors [20]. Retractions are only the tip of the iceberg of problematic papers.

We found that in > 85% of cases, our concerns were confirmed by retraction or EoC, which, in combination with the slow and often absent response, indicates that the number of retractions is not a marker for the true number of problematic studies. We do not think the high combined retraction/EoC rate is inflated, as a few straightforward cases were resolved quicker, while some remain unaddressed. Tables S3A,B describe 6 simple cases from authors of different countries and different journals where, in our opinion, at least an expression of concern should not take more than 2 weeks, but no action has been taken so far.

Others have also raised concerns about the ineffectiveness of the post‐publication assessment [21, 22]. Bolland et al. who reported similar slow response rates to us, proposed a new process for dealing with post‐publication assessment integrity involving the establishment of an independent panel that assesses publication integrity and transparently and timely report the outcomes of those assessments [21].

### 4.4 Interpretation

There is variation between journals. The European Journal of Contraception and Health Care (Taylor and Francis) concluded investigation of 19 papers within 2 years, resulting in 18 retractions [16]. Meanwhile, Contraception (Elsevier) did not solve any of their 11 flagged cases and may not have ever started an investigation. It is unlikely that authors send all their problematic papers to one journal and trustworthy research to others.

The delays we observed matter especially for RCTs, which are included in systematic reviews and meta‐analyses and, via them, determine recommendations in clinical guidelines. Untrustworthy RCT data harms patients, and delay in removing it allows the harm to persist. Recent guidelines on the use of foetal pillow during a Caesarean section were revised after retraction of the RCT, dominating the meta‐analysis 5 years after initial concerns had been raised [23, 24]. In another example, the effectiveness of progesterone for recurrent miscarriage has been called into question following the retractions of large RCTs demonstrating its effectiveness [25, 26, 27]. The American Society for Reproductive Medicine (ASRM) recently decided to exclude an RCT from their thyroid guideline, which reversed previous recommendations to prescribe levothyroxine supplementation in infertile women with subclinical hypothyroidism [28]. The involved journal had published a correction [29]. However, the ASRM practice committee decided to exclude the study. We did not assess this study in the series reported here, but another paper of the same author group received an EoC after we reported signs of data copying [30].

Our findings have implications for publishers. They have both a moral duty to speed up their processes and an interest in doing so. If patients are harmed by treatment based on problematic papers after the publisher has been alerted, publishers could be liable [31]. Delays also affect the public's trust in science. In 2023, a United Kingdom (UK) parliament House of Commons Committee recommended that publishers assess and provide a response within 2 months. This standard is not being met [32].

Publishers might consider the following innovations. Firstly, to use existing manuscript processing software to address post‐publication concerns. This would mean less risk of losing versions, datasets and correspondence than email. Fertility and Sterility have recently introduced an online portal where concerns about potentially problematic papers can be registered directly [33]. Editors should collaborate to review problematic authors who have published across multiple journals. We have published examples of how this might be done [13, 14, 34, 35]. Editors, reviewers and other stakeholders also need training in detecting problematic data because many delays and incorrect decisions are based on ignorance and denial [36]. For some publishers, patient safety and the trustworthiness of science might be of less importance than reputational or legal issues and profit.

Adjustments to the COPE guidelines might help. Current guidelines have no timelines and recommend that the authors' institute(s) should investigate, despite their inherent conflict of interest. Editors and publishers also have a conflict of interest, and a third party evaluating concerns systematically and timely is likely to be an improvement [19]. Issuing an early EoC following the journal's and publisher's initial investigation would allow potentially untrustworthy papers to be publicly flagged prevent the use of the study in guidelines and allow more time for a detailed investigation to be performed without the risk of harm to patients.

Of course, some of the measures proposed above require extra effort and resources, but the alternative—having fake data inform clinical practice—is, at least for us, not acceptable. Also, delayed response results in problematic papers informing meta‐analysis, which requires extra effort to repair [23]. BJOG itself initially refused to investigate an RCT on sildenafil for tocolysis, with the paper being retracted 21 months after the initial concern was raised [37]. This paper is now included in a meta‐analysis suggesting the benefit of sildenafil in pregnancy [38]. Similar to other meta‐analyses, one of which was retracted 5 years and 52 citations after it was published [39, 40]. In reality, sildenafil in pregnancy is not effective and maybe even harmful for the baby [41].

## 5 Conclusion

The publication of untrustworthy papers in scientific literature undermines the credibility and reliability of clinical practice and research. We have highlighted limitations in the current post‐publication review system for problematic papers with both inordinate delays and many investigations never taking place. We urge editors and publishers to work in unison and with reasonable timelines with a priority for patient safety to improve the current post‐publication review system and counteract the rise in false data in scientific literature.

## Author Contributions

The project's aim was discussed and developed with the assistance of B.M.W. and J.T. All authors were involved in data interpretation and revision of the paper. B.W.M., J.T. and J.N. reviewed published papers. B.M.W. and J.T. corresponded with editors and publishers. S.S. screened, verified investigation outcomes, and prepared the manuscript. L.S.A. conducted the statistical analysis. All aspects of the project were conducted with the guidance of BMW.

## Ethics Statement

No ethical approval was required as data was collected from publicly available information.

## Conflicts of Interest

B.W.M. is supported by a National Health Medical Research Council (NHMRC) Practitioner Fellowship (GNT1082548). B.W.M. reports consultancy, travel support and research funding from Merck and consultancy for Organon and Norgine.

## Supporting information


**Table S1.** Supporting Information.

**Table S2.** Supporting Information.

**Data S1.** Supporting Information.

**Data S2.** Supporting Information.

## Data Availability

An anonymous dataset is added to the manuscript.
