# Supplementary material for: Publishers' Response to Post‐Publication Concerns About Clinical Research in Women's Health
Source: BJOG. 2025 Feb 26;132(7):892–901. doi: 10.1111/1471-0528.18100 (PMC12051221; doi:10.1111/1471-0528.18100)
Supplement: Supplementary file 1 — Table S1. Supporting Information. [file BJO-132-892-s001.docx]

**Supplementary Table 1- Journal Case Completion Rate**

| **Status** | | **Q Ran-king** | **Discipline** | **Journal Region** | **Number of Informed Papers** N = 891 | **Case Completion Rate (%)** | **Retraction** N = 152 | **Expression of Concern** N = 75 | **Correction** N = 6 | **Investigation concluded no action** N = 30 | **Pending Investigation** N = 628 | | **Median Time to Response (Months)** *Assuming 30days per month |
| --- | --- | --- | --- | --- | --- | --- | --- | --- | --- | --- | --- | --- | --- |
| Journal of Maternal-Fetal and Neonatal Medicine | | Q2 | Obstetrics and Gynaecology | Europe | 78 (8.8%) | 29 (37%) | 11 (38%) | 18 (62%) | 0 (0%) | 0 (0%) | 49 (63%) | | 28 |
| International Journal of Gynecology & Obstetrics | | Q1 | Obstetrics and Gynaecology | Europe | 67 (7.5%) | 22 (33%) | 12 (57%) | 0 (0%) | 0 (0%) | 10 (43%) | 44 (66%) | 34 | |
| Archives of Gynecology and Obstetrics | | Q2 | Obstetrics and Gynaecology | Europe | 60 (6.7%) | 31 (52%) | 15 (48%) | 16 (52%) | 0 (0%) | 0 (0%) | 29 (48%) | 38 | |
| Fertility and Sterility | | Q1 | Obstetrics and Gynaecology | American | 57 (6.4%) | 21 (37%) | 14 (64%) | 2 (9%) | 1 (5%) | 4 (18%) | 36 (63%) | 31 | |
| European Journal of Obstetrics & Gynecology and Reproductive Biology | | Q2 | Obstetrics and Gynaecology | Europe | 38 (4.3%) | 16 (42%) | 14 (88%) | 2 (12%) | 0 (0%) | 0 (0%) | 22 (58%) | 28 | |
| Gynecological Endocrinology | | Q2 | Obstetrics and Gynaecology | Europe | 27 (3.0%) | 7 (26%) | 5 (71%) | 2 (29%) | 0 (0%) | 0 (0%) | 20 (74%) | 45 | |
| European Journal of Contraception & Reproductive Health Care | | Q2 | Obstetrics and Gynaecology | Europe | 24 (2.7%) | 19 (79%) | 18 (95%) | 0 (0%) | 0 (0%) | 0 (0%) | 6 (25%) | 22 | |
| Journal of Obstetrics and Gynaecology | | Q3 | Obstetrics and Gynaecology | Europe | 23 (2.6%) | 12 (52%) | 12 (100%) | 0 (0%) | 0 (0%) | 0 (0%) | 11 (48%) | 28 | |
| Journal of Obstetrics and Gynaecology Research | | Q2 | Obstetrics and Gynaecology | Oceania | 21 (2.4%) | 4 (19%) | 2 (50%) | 1 (25%) | 1 (25%) | 0 (0%) | 17 (81%) | 42 | |
| Reproductive BioMedicine Online | | Q1 | Obstetrics and Gynaecology | Europe | 20 (2.2%) | 5 (25%) | 5 (100%) | 0 (0%) | 0 (0%) | 0 (0%) | 15 (75%) | 44 | |
| BMC Pregnancy Childbirth | | Q1 | Obstetrics and Gynaecology | Europe | 19 (2.1%) | 6 (32%) | 2 (33%) | 0 (0%) | 2 (33%) | 2 (33%) | 13 (68%) | 13 | |
| Journal of Urololgy | | Q1 | Urology | American | 15 (1.7%) | 14 (93%) | 0 (0%) | 14 (100%) | 0 (0%) | 0 (0%) | 1 (6.7%) | 9 | |
| Journal of Gynecology Obstetrics and Human Reproduction | | Q2 | Obstetrics and Gynaecology | Europe | 13 (1.5%) | 0 (0%) | 0 (0%) | 0 (0%) | 0 (0%) | 0 (0%) | 13 (100%) | N/A | |
| International Journal of Impotence Research | | Q2 | Urology | Europe | 11 (1.2%) | 0 (0%) | 0 (0%) | 0 (0%) | 0 (0%) | 0 (0%) | 11 (100%) | N/A | |
| Journal of Minimally Invasive Gynecology | | Q1 | Obstetrics and Gynaecology | Europe | 11 (1.2%) | 5 (45%) | 0 (0%) | 4 (80%) | 0 (0%) | 1 (30%) | 6 (55%) | 15 | |
| Przeglad Menopauzalny | Q2 | | Obstetrics and Gynaecology | Europe | 11 (1.2%) | 0 (0%) | 0 (0%) | 0 (0%) | 0 (0%) | 0 (0%) | 11 (100%) | N/A | |
| Acta Obstetricia et Gynecologica Scandinavica | Q1 | | Obstetrics and Gynaecology | Europe | 9 (1.0%) | 7 (78%) | 0 (0%) | 6 (86%) | 0 (0%) | 1 (14%) | 2 (22%) | 17 | |
| Gynecologic and Obstetric Investigation | Q2 | | Obstetrics and Gynaecology | Europe | 9 (1.0%) | 7 (78%) | 2 (29%) | 4 (57%) | 0 (0%) | 1 (14%) | 2 (22%) | 8 | |
| Middle East Fertility Society Journal | Q3 | | Obstetrics and Gynaecology | Middle East | 9 (1.0%) | 0 (0%) | 0 (0%) | 0 (0%) | 0 (0%) | 0 (0%) | 9 (100%) | N/A | |
| Reproductive Sciences | Q1 | | Obstetrics and Gynaecology | Europe | 9 (1.0%) | 0 (0%) | 0 (0%) | 0 (0%) | 0 (0%) | 0 (0%) | 9 (100%) | N/A | |
| BJU International | Q1 | | Urology | Europe | 8 (0.9%) | 0 (0%) | 0 (0%) | 0 (0%) | 0 (0%) | 0 (0%) | 8 (100%) | N/A | |
| Hypertension Pregnancy | Q3 | | Obstetrics and Gynaecology | American | 8 (0.9%) | 3 (38%) | 3 (100%) | 0 (0%) | 0 (0%) | 0 (0%) | 5 (63%) | 31 | |
| Evidence Based Women's Health Journal | Unclassified | | Obstetrics and Gynaecology | Middle East | 7 (0.8%) | 0 (0%) | 0 (0%) | 0 (0%) | 0 (0%) | 0 (0%) | 7 (100%) | N/A | |
| Journal of Assisted Reproduction and Genetics | Q1 | | Obstetrics and Gynaecology | American | 7 (0.8%) | 0 (0%) | 0 (0%) | 0 (0%) | 0 (0%) | 0 (0%) | 7 (100%) | N/A | |
| Journal of Perinatal Medicine | Q2 | | Obstetrics and Gynaecology | Europe | 7 (0.8%) | 2 (29%) | 2 (100%) | 0 (0%) | 0 (0%) | 0 (0%) | 5 (71%) | 9 | |
| JBRA Assisted Reproduction | Q2 | | Obstetrics and Gynaecology | South American | 7 (0.8%) | 1 (14%) | 1 (100%) | 0 (0%) | 0 (0%) | 0 (0%) | 6 (86%) | 14 | |
| Pregnancy Hypertension | Q1 | | Obstetrics and Gynaecology | Europe | 7 (0.8%) | 1 (14%) | 0 (0%) | 1 (100%) | 0 (0%) | 0 (0%) | 6 (86%) | 14 | |
| Clinical and Experimental Obstetrics & Gynecology | Q3 | | Obstetrics and Gynaecology | Asia | 6 (0.7%) | 0 (0%) | 0 (0%) | 0 (0%) | 0 (0%) | 0 (0%) | 6 (100%) | N/A | |
| Contraception | Q1 | | Obstetrics and Gynaecology | American | 6 (0.7%) | 1 (17%) | 0 (0%) | 1 (100%) | 0 (0%) | 0 (0%) | 5 (83%) | 8 | |
| Gynecology and Minimally Invasive Therapy | Q3 | | Obstetrics and Gynaecology | Asia | 6 (0.7%) | 0 (0%) | 0 (0%) | 0 (0%) | 0 (0%) | 0 (0%) | 6 (100%) | N/A | |
| Human Reproduction | Q1 | | Obstetrics and Gynaecology | Europe | 6 (0.7%) | 0 (0%) | 0 (0%) | 0 (0%) | 0 (0%) | 0 (0%) | 6 (100%) | N/A | |
| International Urology and Nephrology | Q2 | | Urology | Europe | 6 (0.7%) | 0 (0%) | 0 (0%) | 0 (0%) | 0 (0%) | 0 (0%) | 6 (100%) | N/A | |
| Journal of Obstetrics and Gynecology of India | Q3 | | Obstetrics and Gynaecology | Asia | 6 (0.7%) | 0 (0%) | 0 (0%) | 0 (0%) | 0 (0%) | 0 (0%) | 6 (100%) | N/A | |
| BJOG | Q1 | | Obstetrics and Gynaecology | Europe | 5 (0.6%) | 3 (60%) | 2 (1.3%) | 1 (1.3%) | 0 (0%) | 0 (0%) | 2 (40%) | 18 | |
| European Journal of Gastroenterology & Hepatology | Q3 | | Gastroenterology and Hepatology | American | 5 (0.6%) | 0 (0%) | 0 (0%) | 0 (0%) | 0 (0%) | 0 (0%) | 5 (100%) | N/A | |
| Facts, views & vision in ObGyn | Unclassified | | Obstetrics and Gynaecology | Europe | 5 (0.6%) | 0 (0%) | 0 (0%) | 0 (0%) | 0 (0%) | 0 (0%) | 5 (100%) | N/A | |
| Gynecological Surgery | Q3 | | Obstetrics and Gynaecology | Europe | 5 (0.6%) | 1 (20%) | 1 (100%) | 0 (0%) | 0 (0%) | 0 (0%) | 4 (80%) | N/A | |
| International Journal of Medical Arts | Unclassified | | Unclassified | Middle East | 5 (0.6%) | 0 (0%) | 0 (0%) | 0 (0%) | 0 (0%) | 0 (0%) | 5 (100%) | N/A | |
| Taiwanese Journal of Obstetrics and Gynecology | Q2 | | Obstetrics and Gynaecology | Asia | 5 (0.6%) | 2 (40%) | 0 (0%) | 0 (0%) | 1 (17%) | 1 (3.3%) | 3 (60%) | N/A | |
| Brazilian Journal of Anesthesiolog | Q2 | | Anesthesiology | South American | 5 (0.4%) | 2 (40%) | 2 (100%) | 0 (0%) | 0 (0%) | 0 (0%) | 3 (60%) | 11 | |
| Andrologia | Q3 | | Endocrinology | Europe | 4 (0.4%) | 0 (0%) | 0 (0%) | 0 (0%) | 0 (0%) | 0 (0%) | 4 (100%) | N/A | |
| Cytokine | Q2 | | Multidisciplinary | American | 4 (0.4%) | 4 (100%) | 4 (100%) | 0 (0%) | 0 (0%) | 0 (0%) | 0 (0%) | 20 | |
| European Review for Medical and Pharmacological Sciences | Q2 | | Pharmacology | Europe | 4 (0.4%) | 0 (0%) | 0 (0%) | 0 (0%) | 0 (0%) | 0 (0%) | 4 (100%) | N/A | |
| Journal of Inflammation Research | Q2 | | Immunology | Oceania | 4 (0.4%) | 1 (25%) | 1 (100%) | 0 (0%) | 0 (0%) | 0 (0%) | 3 (75%) | N/A | |
| Journal of Obstetrics and Gynaecology Canada | Q2 | | Obstetrics and Gynaecology | American | 4 (0.4%) | 1 (25%) | 1 (100%) | 0 (0%) | 0 (0%) | 0 (0%) | 3 (75%) | N/A | |
| Journal of the Turkish German Gynecological Association | Q3 | | Obstetrics and Gynaecology | Middle East | 4 (0.4%) | 0 (0%) | 0 (0%) | 0 (0%) | 0 (0%) | 0 (0%) | 4 (100%) | N/A | |
| Minerva Ginecologica | Q3 | | Obstetrics and Gynaecology | Europe | 4 (0.4%) | 0 (0%) | 0 (0%) | 0 (0%) | 0 (0%) | 0 (0%) | 4 (100%) | N/A | |
| Obstetrics and Gynecology | Q1 | | Obstetrics and Gynaecology | American | 4 (0.4%) | 4 (100%) | 1 (25%) | 0 (0%) | 0 (0%) | 3 (75%) | 0 (0%) | 15 | |
| PLoS One | Q1 | | Multidisciplinary | American | 4 (0.4%) | 0 (0%) | 0 (0%) | 0 (0%) | 0 (0%) | 0 (0%) | 4 (100%) | 0 | |
| American Journal of Perinatology | Q2 | | Obstetrics and Gynaecology | American | 3 (0.3%) | 0 (0%) | 0 (0%) | 0 (0%) | 0 (0%) | 0 (0%) | 3 (100%) | N/A | |
| Archives of Virology | Q3 | | Immunology | Europe | 3 (0.3%) | 3 (100%) | 3 (100%) | 0 (0%) | 0 (0%) | 0 (0%) | 0 (0%) | 5 | |
| International Journal of Reproduction, Contraception, Obstetrics and Gynecology | Unclassified | | Obstetrics and Gynaecology | Asia | 3 (0.3%) | 0 (0%) | 0 (0%) | 0 (0%) | 0 (0%) | 0 (0%) | 3 (100%) | N/A | |
| International Journal of Reproductive BioMedicine | Q3 | | Obstetrics and Gynaecology | Middle East | 3 (0.3%) | 0 (0%) | 0 (0%) | 0 (0%) | 0 (0%) | 0 (0%) | 3 (100%) | N/A | |
| Journal of Clinical Psychopharmacology | Q2 | | Pharmacology | American | 3 (0.3%) | 3 (100%) | 3 (100%) | 0 (0%) | 0 (0%) | 0 (0%) | 0 (0%) | 9 | |
| Journal of Reproduction & Infertility | Q3 | | Obstetrics and Gynaecology | Middle East | 3 (0.3%) | 0 (0%) | 0 (0%) | 0 (0%) | 0 (0%) | 0 (0%) | 3 (100%) | N/A | |
| Journal of the Society of Laparoendoscopic Surgeons | Q2 | | Surgery | American | 3 (0.3%) | 0 (0%) | 0 (0%) | 0 (0%) | 0 (0%) | 0 (0%) | 3 (100%) | N/A | |
| Menopause | Q1 | | Obstetrics and Gynaecology | American | 3 (0.3%) | 0 (0%) | 0 (0%) | 0 (0%) | 0 (0%) | 0 (0%) | 3 (100%) | N/A | |
| Al-Azhar International Medical Journal | Unclassified | | Unclassified | Middle East | 2 (0.2%) | 0 (0%) | 0 (0%) | 0 (0%) | 0 (0%) | 0 (0%) | 2 (100%) | N/A | |
| American Journal of Obstetrics and Gynecology | Q1 | | Obstetrics and Gynaecology | American | 2 (0.2%) | 0 (0%) | 0 (0%) | 0 (0%) | 0 (0%) | 0 (0%) | 2 (100%) | N/A | |
| American Journal of Tropical Medicine and Hygiene | Q1 | | Infectious Disease | American | 2 (0.2%) | 2 (100%) | 2 (100%) | 0 (0%) | 0 (0%) | 0 (0%) | 0 (0%) | 12 | |
| Andrology | Q2 | | Endocrinology | American | 2 (0.2%) | 0 (0%) | 0 (0%) | 0 (0%) | 0 (0%) | 0 (0%) | 2 (100%) | N/A | |
| ARC Journal of Gynecology and Obstetrics | Unclassified | | Obstetrics and Gynaecology | Asia | 2 (0.2%) | 0 (0%) | 0 (0%) | 0 (0%) | 0 (0%) | 0 (0%) | 2 (100%) | N/A | |
| Asian Journal of Andrology | Q2 | | Urology | Asia | 2 (0.2%) | 0 (0%) | 0 (0%) | 0 (0%) | 0 (0%) | 0 (0%) | 2 (100%) | N/A | |
| Australian and New Zealand Journal of Obstetrics and Gynaecology | Q2 | | Obstetrics and Gynaecology | American | 2 (0.2%) | 0 (0%) | 0 (0%) | 0 (0%) | 0 (0%) | 0 (0%) | 2 (100%) | N/A | |
| Climacteric | Q2 | | Obstetrics and Gynaecology | Europe | 2 (0.2%) | 0 (0%) | 0 (0%) | 0 (0%) | 0 (0%) | 0 (0%) | 2 (100%) | N/A | |
| Clinical and Applied Thrombosis/Hemostasis | Q2 | | Haematology | American | 2 (0.2%) | 1 (50%) | 0 (0%) | 1 (100%) | 0 (0%) | 0 (0%) | 1 (50%) | 5 | |
| Diabetology & Metabolic Syndrome | Q1 | | Medicine | American | 2 (0.2%) | 0 (0%) | 0 (0%) | 0 (0%) | 0 (0%) | 0 (0%) | 2 (100%) | N/A | |
| Egyptian Journal of Fertility of Sterility | Unclassified | | Obstetrics and Gynaecology | Middle East | 2 (0.2%) | 0 (0%) | 0 (0%) | 0 (0%) | 0 (0%) | 0 (0%) | 2 (100%) | N/A | |
| European Journal of Endocrinology | Q1 | | Endocrinology | Europe | 2 (0.1%) | 1 (100%) | 1 (50%) | 0 (0%) | 0 (0%) | 0 (0%) | 1 (50%) | N/A | |
| European Journal of Pediatrics | Q1 | | Pediatrics | Europe | 2 (0.2%) | 1 (50%) | 0 (0%) | 0 (0%) | 0 (0%) | 1 (50%) | 1 (50%) | N/A | |
| Global Journal of Reproductive Medicine | Unclassified | | Obstetrics and Gynaecology | American | 2 (0.2%) | 0 (0%) | 0 (0%) | 0 (0%) | 0 (0%) | 0 (0%) | 2 (100%) | N/A | |
| Human Fertility | Q2 | | Obstetrics and Gynaecology | Europe | 2 (0.2%) | 0 (0%) | 0 (0%) | 0 (0%) | 0 (0%) | 0 (0%) | 2 (100%) | N/A | |
| International Journal of Obstetrics and Gynaecology Research | Unclassified | | Unclassified | Unclassified | 2 (0.2%) | 0 (0%) | 0 (0%) | 0 (0%) | 0 (0%) | 0 (0%) | 2 (100%) | N/A | |
| International Journal of Fertility and Sterility | Q2 | | Obstetrics and Gynaecology | Middle East | 2 (0.2%) | 0 (0%) | 0 (0%) | 0 (0%) | 0 (0%) | 0 (0%) | 2 (100%) | N/A | |
| International Journal of Obstetric Anesthesia | Q2 | | Obstetrics and Gynaecology | American | 2 (0.2%) | 1 (50%) | 1 (100%) | 0 (0%) | 0 (0%) | 0 (0%) | 1 (50%) | 18 | |
| Journal of Clinical Monitoring and Computing | Q2 | | Anesthesiology | Europe | 2 (0.2%) | 0 (0%) | 0 (0%) | 0 (0%) | 0 (0%) | 0 (0%) | 2 (100%) | N/A | |
| Journal of Clinical and Diagnostic Research | Q4 | | Biochemistry | Asia | 2 (0.2%) | 0 (0%) | 0 (0%) | 0 (0%) | 0 (0%) | 0 (0%) | 2 (100%) | N/A | |
| Journal of Lower Genital Tract Disease | Q1 | | Obstetrics and Gynaecology | American | 2 (0.2%) | 0 (0%) | 0 (0%) | 0 (0%) | 0 (0%) | 0 (0%) | 2 (100%) | N/A | |
| Journal of Perinatology | Q1 | | Obstetrics and Gynaecology | Europe | 2 (0.2%) | 0 (0%) | 0 (0%) | 0 (0%) | 0 (0%) | 0 (0%) | 2 (100%) | N/A | |
| Journal of Tropical Pediatrics | Q2 | | Pediatrics | Europe | 2 (0.2%) | 2 (100%) | 0 (0%) | 0 (0%) | 0 (0%) | 2 (100%) | 0 (0%) | 2 | |
| Journal of Women's Health | Q1 | | Medicine | American | 2 (0.2%) | 0 (0%) | 0 (0%) | 0 (0%) | 0 (0%) | 0 (0%) | 2 (100%) | N/A | |
| Korean Journal of Anesthesiology | Q1 | | Anesthesiology | Asia | 2 (0.2%) | 0 (0%) | 0 (0%) | 0 (0%) | 0 (0%) | 0 (0%) | 2 (100%) | N/A | |
| Obstetrics & Gynecology International Journal | Unclassified | | Obstetrics and Gynaecology | Europe | 2 (0.2%) | 0 (0%) | 0 (0%) | 0 (0%) | 0 (0%) | 0 (0%) | 2 (100%) | N/A | |
| Reproductive Biology and Endocrinology | Q1 | | Obstetrics and Gynaecology | Europe | 2 (0.2%) | 0 (0%) | 0 (0%) | 0 (0%) | 0 (0%) | 0 (0%) | 2 (100%) | N/A | |
| Scientific Reports | Q1 | | Multidisciplinary | Europe | 2 (0.2%) | 1 (50%) | 1 (100%) | 0 (0%) | 0 (0%) | 0 (0%) | 1 (50%) | 2 | |
| American Journal of Obstetrics & Gynecology MFM | Q1 | | Obstetrics and Gynaecology | American | 2 (0.1%) | 0 (0%) | 0 (0%) | 0 (0%) | 0 (0%) | 0 (0%) | 2 (100%) | N/A | |
| Acta Virologica | Q3 | | Infectious Disease | Europe | 1 (0.1%) | 1 (100%) | 0 (0%) | 0 (0%) | 0 (0%) | 1 (100%) | 0 (0%) | 1 | |
| Al-Azhar Medical Journal | Unclassified | | Unclassified | Middle East | 1 (0.1%) | 0 (0%) | 0 (0%) | 0 (0%) | 0 (0%) | 0 (0%) | 1 (100%) | N/A | |
| Anesthesia & Analgesia | Q1 | | Anesthesiology | American | 1 (0.1%) | 0 (0%) | 0 (0%) | 0 (0%) | 0 (0%) | 0 (0%) | 1 (100%) | N/A | |
| Anesthesia Essays and Researches | Unclassified | | Anesthesiology | Asia | 1 (0.1%) | 0 (0%) | 0 (0%) | 0 (0%) | 0 (0%) | 0 (0%) | 1 (100%) | N/A | |
| Annals of Saudi Medicine | Q3 | | Medicine | Middle East | 1 (0.1%) | 0 (0%) | 0 (0%) | 0 (0%) | 0 (0%) | 0 (0%) | 1 (100%) | N/A | |
| Applied Physiology, Nutrition, and Metabolism | Q2 | | Physiology | North American | 1 (0.1%) | 0 (0%) | 0 (0%) | 0 (0%) | 0 (0%) | 0 (0%) | 1 (100%) | N/A | |
| Archives of Osteoporosis | Q2 | | Sport Medicine | Europe | 1 (0.1%) | 0 (0%) | 0 (0%) | 0 (0%) | 0 (0%) | 0 (0%) | 1 (100%) | N/A | |
| Archives of Toxicology | Q1 | | Toxicology | Europe | 1 (0.1%) | 0 (0%) | 0 (0%) | 0 (0%) | 0 (0%) | 0 (0%) | 1 (100%) | N/A | |
| Asian Spine Journal | Q1 | | Surgery | Asia | 1 (0.1%) | 0 (0%) | 0 (0%) | 0 (0%) | 0 (0%) | 0 (0%) | 1 (100%) | N/A | |
| Al-Azhar Assiut Medical Journal | Unclassified | | Unclassified | Middle East | 1 (0.1%) | 0 (0%) | 0 (0%) | 0 (0%) | 0 (0%) | 0 (0%) | 1 (100%) | N/A | |
| Al-Azhar Journal of Pharmaceutical Sciences | Unclassified | | Unclassified | Middle East | 1 (0.1%) | 0 (0%) | 0 (0%) | 0 (0%) | 0 (0%) | 0 (0%) | 1 (100%) | N/A | |
| Biology of Reproduction | Q1 | | Obstetrics and Gynaecology | American | 1 (0.1%) | 1 (100%) | 0 (0%) | 0 (0%) | 0 (0%) | 1 (100%) | 0 (0%) | N/A | |
| Biological Trace Element Research | Q3 | | Multidisciplinary | American | 1 (0.1%) | 1 (100%) | 1 (100%) | 0 (0%) | 0 (0%) | 0 (0%) | 0 (0%) | 28 | |
| Biomedicine and Pharmacotherapy | Q1 | | Pharmacology | Europe | 1 (0.1%) | 0 (0%) | 0 (0%) | 0 (0%) | 0 (0%) | 0 (0%) | 1 (100%) | N/A | |
| Biomedical Research | Unclassified | | Unclassified | Europe | 1 (0.1%) | 0 (0%) | 0 (0%) | 0 (0%) | 0 (0%) | 0 (0%) | 1 (100%) | N/A | |
| BioMed Research International | Q2 | | Biochemistry | American | 1 (0.1%) | 0 (0%) | 0 (0%) | 0 (0%) | 0 (0%) | 0 (0%) | 1 (100%) | N/A | |
| BMC Women's Health | Q2 | | Obstetrics and Gynaecology | Europe | 1 (0.1%) | 0 (0%) | 0 (0%) | 0 (0%) | 0 (0%) | 0 (0%) | 1 (100%) | N/A | |
| BMJ Sexual & Reproductive Health: | Q1 | | Obstetrics and Gynaecology | Europe | 1 (0.1%) | 0 (0%) | 0 (0%) | 0 (0%) | 0 (0%) | 0 (0%) | 1 (100%) | 23 | |
| Cancer Gene Therapy | Q1 | | Biochemistry | Europe | 1 (0.1%) | 1 (100%) | 1 (100%) | 0 (0%) | 0 (0%) | 0 (0%) | 0 (0%) | 3 | |
| Chest | Q1 | | Cardiovascular | American | 1 (0.1%) | 0 (0%) | 0 (0%) | 0 (0%) | 0 (0%) | 0 (0%) | 1 (100%) | N/A | |
| Children (Basel) | Q2 | | Pediatrics | Europe | 1 (0.1%) | 0 (0%) | 0 (0%) | 0 (0%) | 0 (0%) | 0 (0%) | 1 (100%) | N/A | |
| Clinical Medicine Insights | Q3 | | Obstetrics and Gynaecology | American | 1 (0.1%) | 0 (0%) | 0 (0%) | 0 (0%) | 0 (0%) | 0 (0%) | 1 (100%) | N/A | |
| Clinical Nutrition ESPEN | Q2 | | Endocrinology | Europe | 1 (0.1%) | 0 (0%) | 0 (0%) | 0 (0%) | 0 (0%) | 0 (0%) | 1 (100%) | N/A | |
| Clinical Obstetrics, Gynecology and Reproductive Medicine | Unclassified | | Obstetrics and Gynaecology | Europe | 1 (0.1%) | 0 (0%) | 0 (0%) | 0 (0%) | 0 (0%) | 0 (0%) | 1 (100%) | N/A | |
| Clinical Respiratory Journal | Q2 | | Pulmonology | Europe | 1 (0.1%) | 0 (0%) | 0 (0%) | 0 (0%) | 0 (0%) | 0 (0%) | 1 (100%) | N/A | |
| Clinical Research in Obstetrics and Gynecology | Unclassified | | Obstetrics and Gynaecology | American | 1 (0.1%) | 0 (0%) | 0 (0%) | 0 (0%) | 0 (0%) | 0 (0%) | 1 (100%) | N/A | |
| Egyptian Journal of Medical Human Genetic | Q4 | | Genetics | Europe | 1 (0.1%) | 0 (0%) | 0 (0%) | 0 (0%) | 0 (0%) | 0 (0%) | 1 (100%) | N/A | |
| Egyptian Journal of Medical Microbiology | Unclassified | | Unclassified | Middle East | 1 (0.1%) | 0 (0%) | 0 (0%) | 0 (0%) | 0 (0%) | 0 (0%) | 1 (100%) | N/A | |
| Endocrine | Unclassified | | Endocrinology | Unclassified | 1 (0.1%) | 1 (100%) | 0 (0%) | 1 (100%) | 0 (0%) | 0 (0%) | 0 (0%) | 5 | |
| European Journal of Medical Research | Q2 | | Medicine | Europe | 1 (0.1%) | 0 (0%) | 0 (0%) | 0 (0%) | 0 (0%) | 0 (0%) | 1 (100%) | N/A | |
| European Journal of Psychiatry | Q3 | | Psychiatry | Europe | 1 (0.1%) | 0 (0%) | 0 (0%) | 0 (0%) | 0 (0%) | 0 (0%) | 1 (100%) | N/A | |
| Evidence-Based Complementary and Alternative Medicine | Q2 | | Medicine | American | 1 (0.1%) | 0 (0%) | 0 (0%) | 0 (0%) | 0 (0%) | 0 (0%) | 1 (100%) | N/A | |
| Expert Review of Anti-infective Therapy | Q1 | | Infectious Disease | Europe | 1 (0.1%) | 1 (100%) | 1 (100%) | 0 (0%) | 0 (0%) | 0 (0%) | 0 (0%) | 5 | |
| Expert Review of Clinical Pharmacology | Q1 | | Pharmacology | Europe | 1 (0.1%) | 0 (0%) | 0 (0%) | 0 (0%) | 0 (0%) | 0 (0%) | 1 (100%) | N/A | |
| Frontiers in Endocrinology | Q1 | | Endocrinology | Europe | 1 (0.1%) | 0 (0%) | 0 (0%) | 0 (0%) | 0 (0%) | 0 (0%) | 1 (100%) | N/A | |
| Frontiers in Medicine (Lausanne) | Q1 | | Medicine | Europe | 1 (0.1%) | 1 (100%) | 0 (0%) | 0 (0%) | 0 (0%) | 1 (100%) | 0 (0%) | 6 | |
| Frontiers in Pediatrics | Q2 | | Obstetrics and Gynaecology | Europe | 1 (0.1%) | 0 (0%) | 0 (0%) | 0 (0%) | 0 (0%) | 0 (0%) | 1 (100%) | 0 | |
| Geburtshilfe Frauenheilkd | Q3 | | Obstetrics and Gynaecology | Europe | 1 (0.1%) | 1 (100%) | 0 (0%) | 0 (0%) | 1 (100%) | 0 (0%) | 0 (0%) | 13 | |
| Gene Reports | Q4 | | Genetics | American | 1 (0.1%) | 0 (0%) | 0 (0%) | 0 (0%) | 0 (0%) | 0 (0%) | 1 (100%) | N/A | |
| Genetic Testing and Molecular Biomarkers | Q4 | | Genetics | American | 1 (0.1%) | 0 (0%) | 0 (0%) | 0 (0%) | 0 (0%) | 0 (0%) | 1 (100%) | N/A | |
| Gynecology & Reproductive Health | Unclassified | | Obstetrics and Gynaecology | American | 1 (0.1%) | 0 (0%) | 0 (0%) | 0 (0%) | 0 (0%) | 0 (0%) | 1 (100%) | N/A | |
| Gynecology & Obstetrics | Unclassified | | Obstetrics and Gynaecology | Europe | 1 (0.1%) | 0 (0%) | 0 (0%) | 0 (0%) | 0 (0%) | 0 (0%) | 1 (100%) | N/A | |
| Hematology | Q3 | | Haematology | Europe | 1 (0.1%) | 0 (0%) | 0 (0%) | 0 (0%) | 0 (0%) | 0 (0%) | 1 (100%) | N/A | |
| International Journal of Pharmaceutical and Clinical Research i | Q4 | | Pharmacology | Asia | 1 (0.1%) | 0 (0%) | 0 (0%) | 0 (0%) | 0 (0%) | 0 (0%) | 1 (100%) | N/A | |
| Indian Journal of Anaesthesia | Q2 | | Anesthesiology | Asia | 1 (0.1%) | 1 (100%) | 0 (0%) | 0 (0%) | 0 (0%) | 1 (100%) | 0 (0%) | N/A | |
| Indian Journal of Pediatrics | Q2 | | Pediatrics | Asia | 1 (0.1%) | 1 (100%) | 1 (100%) | 0 (0%) | 0 (0%) | 0 (0%) | 0 (0%) | 21 | |
| International Brazilian Journal of Urology | Q2 | | Urology | American | 1 (0.1%) | 0 (0%) | 0 (0%) | 0 (0%) | 0 (0%) | 0 (0%) | 1 (100%) | N/A | |
| International Journal of Childbirth | Q3 | | Obstetrics and Gynaecology | American | 1 (0.1%) | 1 (100%) | 0 (0%) | 1 (100%) | 0 (0%) | 0 (0%) | 0 (0%) | 11 | |
| International Journal of Health Sciences (Qassim) | Unclassified | | Medicine | Middle East | 1 (0.1%) | 0 (0%) | 0 (0%) | 0 (0%) | 0 (0%) | 0 (0%) | 1 (100%) | N/A | |
| International Journal of Occupational and Environmental Hygiene | Unclassified | | Public Health | American | 1 (0.1%) | 0 (0%) | 0 (0%) | 0 (0%) | 0 (0%) | 0 (0%) | 1 (100%) | N/A | |
| International Journal of Occupational and Environmental Medicine | Q2 | | Public Health | Middle East | 1 (0.1%) | 0 (0%) | 0 (0%) | 0 (0%) | 0 (0%) | 0 (0%) | 1 (100%) | N/A | |
| International Journal of Gynecology, Obstetrics and Neonatal Care, | Unclassified | | Obstetrics and Gynaecology | Asia | 1 (0.1%) | 0 (0%) | 0 (0%) | 0 (0%) | 0 (0%) | 0 (0%) | 1 (100%) | N/A | |
| International Urogynecology Journal | Q2 | | Obstetrics and Gynaecology | Europe | 1 (0.1%) | 0 (0%) | 0 (0%) | 0 (0%) | 0 (0%) | 0 (0%) | 1 (100%) | N/A | |
| Italian Journal of Gynaecology & Obstetrics | Q3 | | Obstetrics and Gynaecology | Europe | 1 (0.1%) | 0 (0%) | 0 (0%) | 0 (0%) | 0 (0%) | 0 (0%) | 1 (100%) | N/A | |
| Journal of Advanced Nutritional and Human Metabolism | Unclassified | | Unclassified | American | 1 (0.1%) | 0 (0%) | 0 (0%) | 0 (0%) | 0 (0%) | 0 (0%) | 1 (100%) | N/A | |
| Journal of Basic and Clinical Reproductive Sciences | Unclassified | | Unclassified | Africa | 1 (0.1%) | 0 (0%) | 0 (0%) | 0 (0%) | 0 (0%) | 0 (0%) | 1 (100%) | N/A | |
| Journal of Clinical Gastroenterology | Q2 | | Gastroenterology and Hepatology | American | 1 (0.1%) | 0 (0%) | 0 (0%) | 0 (0%) | 0 (0%) | 0 (0%) | 1 (100%) | N/A | |
| Journal of Bone and Mineral Metabolism | Q2 | | Orthopedics | Asia | 1 (0.1%) | 0 (0%) | 0 (0%) | 0 (0%) | 0 (0%) | 0 (0%) | 1 (100%) | N/A | |
| Journal of Clinical Gynecology and Obstetrics | Unclassified | | Obstetrics and Gynaecology | American | 1 (0.1%) | 0 (0%) | 0 (0%) | 0 (0%) | 0 (0%) | 0 (0%) | 1 (100%) | N/A | |
| Journal of Clinical Periodontology | Q1 | | Periodontics | Europe | 1 (0.1%) | 0 (0%) | 0 (0%) | 0 (0%) | 0 (0%) | 0 (0%) | 1 (100%) | N/A | |
| Journal of Clinical Pharmacy and Therapeutics | Q3 | | Pharmacology | Europe | 1 (0.1%) | 0 (0%) | 0 (0%) | 0 (0%) | 0 (0%) | 0 (0%) | 1 (100%) | N/A | |
| Journal of Clinical Ultrasound | Q3 | | Radiology | American | 1 (0.1%) | 0 (0%) | 0 (0%) | 0 (0%) | 0 (0%) | 0 (0%) | 1 (100%) | N/A | |
| Journal of Diabetes and its Complications | Q2 | | Endocrinology | American | 1 (0.1%) | 0 (0%) | 0 (0%) | 0 (0%) | 0 (0%) | 0 (0%) | 1 (100%) | N/A | |
| Journal of Endocrinology | Q1 | | Endocrinology | Europe | 1 (0.1%) | 1 (100%) | 1 (100%) | 0 (0%) | 0 (0%) | 0 (0%) | 0 (0%) | 9 | |
| Journal of the Egyptian Public Health Association | Q2 | | Public Health | Europe | 1 (0.1%) | 0 (0%) | 0 (0%) | 0 (0%) | 0 (0%) | 0 (0%) | 1 (100%) | N/A | |
| Journal of Family and Reproductive Health | Q1 | | Obstetrics and Gynaecology | Middle East | 1 (0.1%) | 0 (0%) | 0 (0%) | 0 (0%) | 0 (0%) | 0 (0%) | 1 (100%) | N/A | |
| Journal of Genetic Engineering and Biotechnology | Q2 | | Genetics | Europe | 1 (0.1%) | 0 (0%) | 0 (0%) | 0 (0%) | 0 (0%) | 0 (0%) | 1 (100%) | N/A | |
| Journal of Gynecology and Obstetrics Bulletin | Unclassified | | Obstetrics and Gynaecology | Europe | 1 (0.1%) | 0 (0%) | 0 (0%) | 0 (0%) | 0 (0%) | 0 (0%) | 1 (100%) | N/A | |
| Journal of Gynecologic Oncology | Q1 | | Obstetrics and Gynaecology | Asia | 1 (0.1%) | 0 (0%) | 0 (0%) | 0 (0%) | 0 (0%) | 0 (0%) | 1 (100%) | N/A | |
| Journal Of Herbal Pharmacotherapy | Q4 | | Pharmacology | American | 1 (0.1%) | 0 (0%) | 0 (0%) | 0 (0%) | 0 (0%) | 0 (0%) | 1 (100%) | N/A | |
| Journal of Hospital Infection | Q1 | | Infectious Disease | Europe | 1 (0.1%) | 0 (0%) | 0 (0%) | 0 (0%) | 0 (0%) | 0 (0%) | 1 (100%) | N/A | |
| Journal of Human Genetics | Q2 | | Genetics | Europe | 1 (0.1%) | 0 (0%) | 0 (0%) | 0 (0%) | 0 (0%) | 0 (0%) | 1 (100%) | N/A | |
| Journal of Medical Virology | Q1 | | Immunology | American | 1 (0.1%) | 0 (0%) | 0 (0%) | 0 (0%) | 0 (0%) | 0 (0%) | 1 (100%) | N/A | |
| Journal of Mid-life Health | Q3 | | Medicine | Asia | 1 (0.1%) | 1 (100%) | 1 (100%) | 0 (0%) | 0 (0%) | 0 (0%) | 0 (0%) | 16 | |
| Journal of Obstetrics and Gynecology: Open Access | Unclassified | | Obstetrics and Gynaecology | American | 1 (0.1%) | 0 (0%) | 0 (0%) | 0 (0%) | 0 (0%) | 0 (0%) | 1 (100%) | N/A | |
| Journal of Pain & Relief | Unclassified | | Anesthesiology | American | 1 (0.1%) | 0 (0%) | 0 (0%) | 0 (0%) | 0 (0%) | 0 (0%) | 1 (100%) | N/A | |
| Journal of the Pakistan Medical Association | Q4 | | Medicine | Asia | 1 (0.1%) | 0 (0%) | 0 (0%) | 0 (0%) | 0 (0%) | 0 (0%) | 1 (100%) | N/A | |
| Journal of Pediatric and Adolescent Gynecology | Q2 | | Obstetrics and Gynaecology | American | 1 (0.1%) | 0 (0%) | 0 (0%) | 0 (0%) | 0 (0%) | 0 (0%) | 1 (100%) | N/A | |
| Journal of Pediatric Urology | Q2 | | Pediatrics | Europe | 1 (0.1%) | 1 (100%) | 1 (100%) | 0 (0%) | 0 (0%) | 0 (0%) | 0 (0%) | 9 | |
| Journal of Pregnancy and Child Health | Unclassified | | Obstetrics and Gynaecology | American | 1 (0.1%) | 0 (0%) | 0 (0%) | 0 (0%) | 0 (0%) | 0 (0%) | 1 (100%) | N/A | |
| Journal of Psychosomatic Obstetrics & Gynecology | Q1 | | Psychiatry | American | 1 (0.1%) | 0 (0%) | 0 (0%) | 0 (0%) | 0 (0%) | 0 (0%) | 1 (100%) | N/A | |
| Journal of Reproduction and Contraception | Q4 | | Medicine | Asia | 1 (0.1%) | 0 (0%) | 0 (0%) | 0 (0%) | 0 (0%) | 0 (0%) | 1 (100%) | N/A | |
| Journal of Sexual Medicine | Q1 | | Obstetrics and Gynaecology | Europe | 1 (0.1%) | 0 (0%) | 0 (0%) | 0 (0%) | 0 (0%) | 0 (0%) | 1 (100%) | N/A | |
| Journal of the Society for Gynecologic Investigation | Unclassified | | Obstetrics and Gynaecology | Europe | 1 (0.1%) | 0 (0%) | 0 (0%) | 0 (0%) | 0 (0%) | 0 (0%) | 1 (100%) | N/A | |
| Journal of Steroid Biochemistry and Molecular Biology | Q2 | | Multidisciplinary | Europe | 1 (0.1%) | 0 (0%) | 0 (0%) | 0 (0%) | 0 (0%) | 0 (0%) | 1 (100%) | N/A | |
| Journal of Strength & Conditioning Research | Q1 | | Physiology | American | 1 (0.1%) | 0 (0%) | 0 (0%) | 0 (0%) | 0 (0%) | 0 (0%) | 1 (100%) | N/A | |
| Journal of Clinical Endocrinology and Metabolism | Q1 | | Endocrinology | American | 1 (0.1%) | 0 (0%) | 0 (0%) | 0 (0%) | 0 (0%) | 0 (0%) | 1 (100%) | N/A | |
| Knowledge Commons | Unclassified | | Medicine | American | 1 (0.1%) | 0 (0%) | 0 (0%) | 0 (0%) | 0 (0%) | 0 (0%) | 1 (100%) | N/A | |
| Life Sciences | Q1 | | Multidisciplinary | American | 1 (0.1%) | 0 (0%) | 0 (0%) | 0 (0%) | 0 (0%) | 0 (0%) | 1 (100%) | N/A | |
| Liver International | Q1 | | Gastroenterology and Hepatology | Europe | 1 (0.1%) | 0 (0%) | 0 (0%) | 0 (0%) | 0 (0%) | 0 (0%) | 1 (100%) | N/A | |
| Mathews Journal of Gynecology and Obstetrics | Q1 | | Obstetrics and Gynaecology | American | 1 (0.1%) | 0 (0%) | 0 (0%) | 0 (0%) | 0 (0%) | 0 (0%) | 1 (100%) | N/A | |
| Maturitas | Q1 | | Obstetrics and Gynaecology | Europe | 1 (0.1%) | 0 (0%) | 0 (0%) | 0 (0%) | 0 (0%) | 0 (0%) | 1 (100%) | N/A | |
| Medical Archives | Unclassified | | Medicine | Europe | 1 (0.1%) | 0 (0%) | 0 (0%) | 0 (0%) | 0 (0%) | 0 (0%) | 1 (100%) | N/A | |
| Medical & Biological Engineering & Computing | Q2 | | Biomedical Engineering | Europe | 1 (0.1%) | 0 (0%) | 0 (0%) | 0 (0%) | 0 (0%) | 0 (0%) | 1 (100%) | N/A | |
| Medical Oncology | Q2 | | Oncology | Europe | 1 (0.1%) | 0 (0%) | 0 (0%) | 0 (0%) | 0 (0%) | 0 (0%) | 1 (100%) | N/A | |
| Menoufia Medical Journal | Unclassified | | Medicine | Middle East | 1 (0.1%) | 0 (0%) | 0 (0%) | 0 (0%) | 0 (0%) | 0 (0%) | 1 (100%) | N/A | |
| Minerva Chirurgica | Q3 | | Surgery | Europe | 1 (0.1%) | 1 (100%) | 1 (100%) | 0 (0%) | 0 (0%) | 0 (0%) | 0 (0%) | 6 | |
| Neuropsychopharmacology | Q1 | | Pharmacology | Europe | 1 (0.1%) | 1 (100%) | 1 (100%) | 0 (0%) | 0 (0%) | 0 (0%) | 0 (0%) | 10 | |
| Journal of Neurosciences in Rural Practice | Q3 | | Neurology | Asia | 1 (0.1%) | 0 (0%) | 0 (0%) | 0 (0%) | 0 (0%) | 0 (0%) | 1 (100%) | 18 | |
| Nutrients | Q1 | | Nutrition | Europe | 1 (0.1%) | 0 (0%) | 0 (0%) | 0 (0%) | 0 (0%) | 0 (0%) | 1 (100%) | N/A | |
| Obesity Research & Clinical Practice | Q2 | | Endocrinology | Europe | 1 (0.1%) | 0 (0%) | 0 (0%) | 0 (0%) | 0 (0%) | 0 (0%) | 1 (100%) | N/A | |
| Obstetrics and Gynecology International | Q3 | | Obstetrics and Gynaecology | American | 1 (0.1%) | 0 (0%) | 0 (0%) | 0 (0%) | 0 (0%) | 0 (0%) | 1 (100%) | N/A | |
| Obstetrics & Gynecology Science | Q2 | | Obstetrics and Gynaecology | Asia | 1 (0.1%) | 0 (0%) | 0 (0%) | 0 (0%) | 0 (0%) | 0 (0%) | 1 (100%) | N/A | |
| Journal of Obstetrics Gynecology and Reproductive Sciences | Unclassified | | Obstetrics and Gynaecology | American | 1 (0.1%) | 0 (0%) | 0 (0%) | 0 (0%) | 0 (0%) | 0 (0%) | 1 (100%) | N/A | |
| Pakistan Journal of Biological Sciences | Q3 | | Biology | Asia | 1 (0.1%) | 0 (0%) | 0 (0%) | 0 (0%) | 0 (0%) | 0 (0%) | 1 (100%) | N/A | |
| Pediatric Research | Q1 | | Pediatrics | American | 1 (0.1%) | 0 (0%) | 0 (0%) | 0 (0%) | 0 (0%) | 0 (0%) | 1 (100%) | N/A | |
| Pediatric Nephrology | Q2 | | Pediatrics | Europe | 1 (0.1%) | 0 (0%) | 0 (0%) | 0 (0%) | 0 (0%) | 0 (0%) | 1 (100%) | N/A | |
| Phytotherapy Research | Q1 | | Pharmacology | Europe | 1 (0.1%) | 0 (0%) | 0 (0%) | 0 (0%) | 0 (0%) | 0 (0%) | 1 (100%) | N/A | |
| Postgraduate Medical Journal | Q1 | | Medicine | Europe | 1 (0.1%) | 0 (0%) | 0 (0%) | 0 (0%) | 0 (0%) | 0 (0%) | 1 (100%) | N/A | |
| Proceedings in Obstetrics Gynecology | Unclassified | | Obstetrics and Gynaecology | American | 1 (0.1%) | 0 (0%) | 0 (0%) | 0 (0%) | 0 (0%) | 0 (0%) | 1 (100%) | N/A | |
| QJM: An International Journal of Medicine | Q2 | | Medicine | Europe | 1 (0.1%) | 0 (0%) | 0 (0%) | 0 (0%) | 0 (0%) | 0 (0%) | 1 (100%) | N/A | |
| Reproductive Health | Q1 | | Obstetrics and Gynaecology | Europe | 1 (0.1%) | 0 (0%) | 0 (0%) | 0 (0%) | 0 (0%) | 0 (0%) | 1 (100%) | N/A | |
| Reproductive Toxicology | Q2 | | Toxicology | American | 1 (0.1%) | 0 (0%) | 0 (0%) | 0 (0%) | 0 (0%) | 0 (0%) | 1 (100%) | N/A | |
| Reproduction | Q1 | | Obstetrics and Gynaecology | Europe | 1 (0.1%) | 0 (0%) | 0 (0%) | 0 (0%) | 0 (0%) | 0 (0%) | 1 (100%) | N/A | |
| SunKrist Journal of Obstetrics and Gynecology Research | Unclassified | | Obstetrics and Gynaecology | Unclassified | 1 (0.1%) | 0 (0%) | 0 (0%) | 0 (0%) | 0 (0%) | 0 (0%) | 1 (100%) | N/A | |
| Surgical Laparoscopy, Endoscopy, and Percutaneous Techniques | Q2 | | Surgery | American | 1 (0.1%) | 0 (0%) | 0 (0%) | 0 (0%) | 0 (0%) | 0 (0%) | 1 (100%) | N/A | |
| Ultrasound in Medicine and Biology | Q2 | | Radiology | American | 1 (0.1%) | 0 (0%) | 0 (0%) | 0 (0%) | 0 (0%) | 0 (0%) | 1 (100%) | N/A | |
| Ultrasound in Obstetrics & Gynecology | Q1 | | Radiology | Europe | 1 (0.1%) | 0 (0%) | 0 (0%) | 0 (0%) | 0 (0%) | 0 (0%) | 1 (100%) | N/A | |
| Urolithiasis | Q1 | | Urology | Europe | 1 (0.1%) | 0 (0%) | 0 (0%) | 0 (0%) | 0 (0%) | 0 (0%) | 1 (100%) | N/A | |
| Vascular Pharmacology | Q1 | | Pharmacology | American | 1 (0.1%) | 1 (100%) | 1 (100%) | 0 (0%) | 0 (0%) | 0 (0%) | 0 (0%) | 9 | |
| % Calculated from the number of completed cases | | | | | | | | | | | | | |
