# Supplementary material for: Publishers' Response to Post‐Publication Concerns About Clinical Research in Women's Health
Source: BJOG. 2025 Feb 26;132(7):892–901. doi: 10.1111/1471-0528.18100 (PMC12051221; doi:10.1111/1471-0528.18100)
Supplement: Supplementary file 2 — Table S2. Supporting Information. [file BJO-132-892-s003.docx]

**Supplementary Table 2 – Publisher Case Completion Rate**

| **Status** | **Number of Informed Papers** N = 891 | **Case Completion Rate (%)** | **Retraction** N = 152 | **Expression of Concern** N = 75 | **Correction** N = 6 | **Investigation concluded no action** N = 30 | **Pending Investigation** N = 628 | **Median Time to Response (Days)** | **Median Time to Response (Months)** *Assuming 30days per month |
| --- | --- | --- | --- | --- | --- | --- | --- | --- | --- |
| Elsevier | 204 (23%) | 61 (30%) | 43 (70%) | 10 (16%) | 2 (3%) | 6 (10%) | 143 (70%) | 518 | 42 |
| Taylor Francis | 170 (19%) | 70 (41%) | 50 (71%) | 20 (28%) | 0 (0%) | 0 (0%) | 100 (59%) | 610 | 28 |
| Springer | 154 (17%) | 50 (33%) | 26 (52%) | 18 (36%) | 2 (4%) | 4 (8%) | 103 (67%) | 530 | 34 |
| Wiley Blackwell | 125 (14%) | 37 (30%) | 17 (46%) | 8 (22%) | 1 (3%) | 11 (30%) | 88 (70%) | 711 | 34 |
| Wolters Kluwer | 45 (5.1%) | 22 (49%) | 5 (23%) | 14 (63%) | 0 (0%) | 3 (14%) | 23 (51%) | 398 | 18 |
| Oxford University Press | 13 (1.5%) | 3 (23%) | 1 (33%) | 0 (0%) | 0 (0%) | 2 (67%) | 10 (77%) | 293 | N/A |
| Termedia | 11 (1.2%) | 0 (0%) | 0 (0%) | 0 (0%) | 0 (0%) | 0 (0%) | 11 (100%) | 521 | N/A |
| Karger | 9 (1.0%) | 7 (78%) | 2 (29%) | 4 (57%) | 0 (0%) | 1 (14%) | 2 (22%) | 164 | 7 |
| Al-Azhar University | 8 (0.9%) | 0 (0%) | 0 (0%) | 0 (0%) | 0 (0%) | 0 (0%) | 8 (100%) | 144 | N/A |
| De Gruyter | 7 (0.8%) | 2 (29%) | 2 (100%) | 0 (0%) | 0 (0%) | 0 (0%) | 5 (71%) | 541 | N/A |
| Brazilian Society of Assisted Reproduction | 7 (0.8%) | 1 (14%) | 1 (100%) | 0 (0%) | 0 (0%) | 0 (0%) | 6 (86%) | 416 | N/A |
| Evidence Based Women's Health Society | 6 (0.7%) | 0 (0%) | 0 (0%) | 0 (0%) | 0 (0%) | 0 (0%) | 6 (100%) | 565 | N/A |
| IMR Press | 6 (0.7%) | 0 (0%) | 0 (0%) | 0 (0%) | 0 (0%) | 0 (0%) | 6 (100%) | 788 | N/A |
| BioMed Central | 5 (0.6%) | 0 (0%) | 0 (0%) | 0 (0%) | 0 (0%) | 0 (0%) | 5 (100%) | 92 | N/A |
| Edizioni Minerva Medica | 5 (0.6%) | 1 (50%) | 1 (50%) | 0 (0%) | 0 (0%) | 0 (0%) | 4 (80%) | 310 | N/A |
| Flemish Society of Obstetrics and Gynecology | 5 (0.6%) | 0 (0%) | 0 (0%) | 0 (0%) | 0 (0%) | 0 (0%) | 5 (100%) | 470 | N/A |
| Dove Medical Press | 4 (0.4%) | 1 (25%) | 1 (100%) | 0 (0%) | 0 (0%) | 0 (0%) | 3 (75%) | 737 | N/A |
| Galenos | 4 (0.4%) | 0 (0%) | 0 (0%) | 0 (0%) | 0 (0%) | 0 (0%) | 4 (100%) | 506 | N/A |
| PLoS | 4 (0.4%) | 0 (0%) | 0 (0%) | 0 (0%) | 0 (0%) | 0 (0%) | 4 (100%) | 236 | N/A |
| Thieme | 4 (0.4%) | 1 (25%) | 0 (0%) | 0 (0%) | 1 (100%) | 0 (0%) | 3 (75%) | 649 | N/A |
| Veduci Editore | 4 (0.4%) | 0 (0%) | 0 (0%) | 0 (0%) | 0 (0%) | 0 (0%) | 4 (100%) | 181 | N/A |
| Avicenna Research Institute | 3 (0.3%) | 0 (0%) | 0 (0%) | 0 (0%) | 0 (0%) | 0 (0%) | 3 (100%) | 723 | N/A |
| Frontiers media | 3 (0.3%) | 1 (33%) | 0 (0%) | 0 (0%) | 0 (0%) | 1 (100%) | 2 (67%) | 183 | N/A |
| Gavin Publishers | 2 (0.2%) | 0 (0%) | 0 (0%) | 0 (0%) | 0 (0%) | 0 (0%) | 2 (100%) | 365 | N/A |
| Hindawi | 3 (0.3%) | 0 (0%) | 0 (0%) | 0 (0%) | 0 (0%) | 0 (0%) | 3 (100%) | 717 | N/A |
| KNE Publishing | 3 (0.3%) | 0 (0%) | 0 (0%) | 0 (0%) | 0 (0%) | 0 (0%) | 3 (100%) | 450 | N/A |
| Mary Ann Liebert Inc | 3 (0.3%) | 0 (0%) | 0 (0%) | 0 (0%) | 0 (0%) | 0 (0%) | 3 (100%) | 760 | N/A |
| Medip Academy | 3 (0.3%) | 0 (0%) | 0 (0%) | 0 (0%) | 0 (0%) | 0 (0%) | 3 (100%) | 365 | N/A |
| Medknow Publications | 3 (0.3%) | 1 (33%) | 0 (0%) | 0 (0%) | 0 (0%) | 1 (100%) | 2 (67%) | 710 | N/A |
| Sage | 3 (0.3%) | 1 (33%) | 0 (0%) | 1 (100%) | 0 (0%) | 0 (0%) | 2 (67%) | 147 | N/A |
| Society of Laparoscopic and Robotic Surgery | 3 (0.3%) | 0 (0%) | 0 (0%) | 0 (0%) | 0 (0%) | 0 (0%) | 3 (100%) | 750 | N/A |
| ARC Publications | 2 (0.2%) | 0 (0%) | 0 (0%) | 0 (0%) | 0 (0%) | 0 (0%) | 2 (100%) | 989 | N/A |
| American Society of Tropical Medicine and Hygiene | 2 (0.2%) | 2 (100%) | 2 (100%) | 0 (0%) | 0 (0%) | 0 (0%) | 0 (0%) | 348 | N/A |
| Bioscientifica | 2 (0.2%) | 1 (50%) | 1 (100%) | 0 (0%) | 0 (0%) | 0 (0%) | 1 (50%) | 510 | N/A |
| Egyptian Fertility and Sterility Society | 2 (0.2%) | 0 (0%) | 0 (0%) | 0 (0%) | 0 (0%) | 0 (0%) | 2 (100%) | 676 | 23 |
| International Journal of Obstetrics and Gynaecology Research Publications | 2 (0.2%) | 0 (0%) | 0 (0%) | 0 (0%) | 0 (0%) | 0 (0%) | 2 (100%) | 365 | N/A |
| Journal of Clinical and Diagnostic Research Pre-Publishing | 2 (0.2%) | 0 (0%) | 0 (0%) | 0 (0%) | 0 (0%) | 0 (0%) | 2 (100%) | 995 | N/A |
| Juniper Publishers | 2 (0.2%) | 0 (0%) | 0 (0%) | 0 (0%) | 0 (0%) | 0 (0%) | 2 (100%) | 631 | N/A |
| Korean Society of Anesthesiologists | 2 (0.2%) | 0 (0%) | 0 (0%) | 0 (0%) | 0 (0%) | 0 (0%) | 2 (100%) | 745 | N/A |
| MDPI | 2 (0.2%) | 0 (0%) | 0 (0%) | 0 (0%) | 0 (0%) | 0 (0%) | 2 (100%) | 164 | N/A |
| MedCrave | 2 (0.2%) | 0 (0%) | 0 (0%) | 0 (0%) | 0 (0%) | 0 (0%) | 2 (100%) | 741 | N/A |
| OMICS | 2 (0.2%) | 0 (0%) | 0 (0%) | 0 (0%) | 0 (0%) | 0 (0%) | 2 (100%) | 365 | N/A |
| Royan Institute of Iran | 2 (0.2%) | 0 (0%) | 0 (0%) | 0 (0%) | 0 (0%) | 0 (0%) | 2 (100%) | 713 | N/A |
| Shanghai Materia Medica | 2 (0.2%) | 0 (0%) | 0 (0%) | 0 (0%) | 0 (0%) | 0 (0%) | 2 (100%) | 390 | N/A |
| Academy of Medical Sciences of Bosnia and Herzegovina | 1 (0.1%) | 0 (0%) | 0 (0%) | 0 (0%) | 0 (0%) | 0 (0%) | 1 (100%) | 749 | N/A |
| Academic Publishing and Translation | 1 (0.1%) | 0 (0%) | 0 (0%) | 0 (0%) | 0 (0%) | 0 (0%) | 1 (100%) | 761 | N/A |
| Al-Azhar Scientific Medical Society | 1 (0.1%) | 0 (0%) | 0 (0%) | 0 (0%) | 0 (0%) | 0 (0%) | 1 (100%) | 144 | N/A |
| Allied academies | 1 (0.1%) | 0 (0%) | 0 (0%) | 0 (0%) | 0 (0%) | 0 (0%) | 1 (100%) | 777 | N/A |
| Asian Network for Scientific Information | 1 (0.1%) | 0 (0%) | 0 (0%) | 0 (0%) | 0 (0%) | 0 (0%) | 1 (100%) | 717 | N/A |
| Ascepius | 1 (0.1%) | 0 (0%) | 0 (0%) | 0 (0%) | 0 (0%) | 0 (0%) | 1 (100%) | 146 | N/A |
| Asian Society of Gynecologic Oncology | 1 (0.1%) | 0 (0%) | 0 (0%) | 0 (0%) | 0 (0%) | 0 (0%) | 1 (100%) | 779 | N/A |
| Auctores Publishing | 1 (0.1%) | 0 (0%) | 0 (0%) | 0 (0%) | 0 (0%) | 0 (0%) | 1 (100%) | 881 | N/A |
| BMJ Publishing | 1 (0.1%) | 0 (0%) | 0 (0%) | 0 (0%) | 0 (0%) | 0 (0%) | 1 (100%) | 680 | N/A |
| Brazilian Society of Urology | 1 (0.1%) | 0 (0%) | 0 (0%) | 0 (0%) | 0 (0%) | 0 (0%) | 1 (100%) | 293 | N/A |
| Canadaian Science Publishing | 1 (0.1%) | 0 (0%) | 0 (0%) | 0 (0%) | 0 (0%) | 0 (0%) | 1 (100%) | 756 | N/A |
| Churchill Livingstone | 1 (0.1%) | 0 (0%) | 0 (0%) | 0 (0%) | 0 (0%) | 0 (0%) | 1 (100%) | 710 | N/A |
| Cosmos Scholars Publishing House | 1 (0.1%) | 0 (0%) | 0 (0%) | 0 (0%) | 0 (0%) | 0 (0%) | 1 (100%) | 539 | N/A |
| Elmer Press | 1 (0.1%) | 0 (0%) | 0 (0%) | 0 (0%) | 0 (0%) | 0 (0%) | 1 (100%) | 796 | N/A |
| Endocrine Society | 1 (0.1%) | 0 (0%) | 0 (0%) | 0 (0%) | 0 (0%) | 0 (0%) | 1 (100%) | 1312 | N/A |
| Egyptian Society for Medical Microbiology | 1 (0.1%) | 0 (0%) | 0 (0%) | 0 (0%) | 0 (0%) | 0 (0%) | 1 (100%) | 674 | N/A |
| Czechoslovak Academy of Sciences | 1 (0.1%) | 1 (100%) | 0 (0%) | 0 (0%) | 0 (0%) | 1 (100%) | 0 (0%) | 35 | N/A |
| University of Iowa Hospitals and Clinics | 1 (0.1%) | 0 (0%) | 0 (0%) | 0 (0%) | 0 (0%) | 0 (0%) | 1 (100%) | 410 | N/A |
| Dr. Yashwant Research Labs Pvt. Ltd | 1 (0.1%) | 0 (0%) | 0 (0%) | 0 (0%) | 0 (0%) | 0 (0%) | 1 (100%) | 154 | N/A |
| King Faisal Specialist Hospital and Research Center and King Saud University College of Medicine | 1 (0.1%) | 0 (0%) | 0 (0%) | 0 (0%) | 0 (0%) | 0 (0%) | 1 (100%) | 741 | N/A |
| Korean Society of Obstetrics and Gynecology | 1 (0.1%) | 0 (0%) | 0 (0%) | 0 (0%) | 0 (0%) | 0 (0%) | 1 (100%) | 43 | N/A |
| Korean Society of Spine Surgery | 1 (0.1%) | 0 (0%) | 0 (0%) | 0 (0%) | 0 (0%) | 0 (0%) | 1 (100%) | 554 | N/A |
| Longdom Publishing SL | 2 (0.2%) | 0 (0%) | 0 (0%) | 0 (0%) | 0 (0%) | 0 (0%) | 2 (100%) | 554 | N/A |
| Mathews International LLC | 1 (0.1%) | 0 (0%) | 0 (0%) | 0 (0%) | 0 (0%) | 0 (0%) | 1 (100%) | 776 | N/A |
| NIOC Health Organization | 1 (0.1%) | 0 (0%) | 0 (0%) | 0 (0%) | 0 (0%) | 0 (0%) | 1 (100%) | 732 | N/A |
| Open Access Text | 1 (0.1%) | 0 (0%) | 0 (0%) | 0 (0%) | 0 (0%) | 0 (0%) | 1 (100%) | 792 | N/A |
| Pakistan Medical Association | 1 (0.1%) | 0 (0%) | 0 (0%) | 0 (0%) | 0 (0%) | 0 (0%) | 1 (100%) | 726 | N/A |
| Population Council | 1 (0.1%) | 0 (0%) | 0 (0%) | 0 (0%) | 0 (0%) | 0 (0%) | 1 (100%) | 69 | N/A |
| Scientific Scholar | 1 (0.1%) | 0 (0%) | 0 (0%) | 0 (0%) | 0 (0%) | 0 (0%) | 1 (100%) | 554 | N/A |
| Scivision | 1 (0.1%) | 0 (0%) | 0 (0%) | 0 (0%) | 0 (0%) | 0 (0%) | 1 (100%) | 365 | N/A |
| Edra S.p.A | 1 (0.1%) | 0 (0%) | 0 (0%) | 0 (0%) | 0 (0%) | 0 (0%) | 1 (100%) | 146 | N/A |
| Society of Reproductive Biologists of Nigeria | 1 (0.1%) | 0 (0%) | 0 (0%) | 0 (0%) | 0 (0%) | 0 (0%) | 1 (100%) | 927 | N/A |
| SunKrist Publishing | 1 (0.1%) | 0 (0%) | 0 (0%) | 0 (0%) | 0 (0%) | 0 (0%) | 1 (100%) | 928 | N/A |
| Tehran University of Medical Sciences | 1 (0.1%) | 0 (0%) | 0 (0%) | 0 (0%) | 0 (0%) | 0 (0%) | 1 (100%) | 896 | N/A |
| Verizona Publisher | 1 (0.1%) | 0 (0%) | 0 (0%) | 0 (0%) | 0 (0%) | 0 (0%) | 1 (100%) | 365 | N/A |
| W.B. Saunders | 1 (0.1%) | 0 (0%) | 0 (0%) | 0 (0%) | 0 (0%) | 0 (0%) | 1 (100%) | 700 | N/A |
| % Calculated from the number of completed cases | | | | | | | | | |
|  |  |  |  |  |  |  |  |  |  |
