# Supplementary material for: Publishers' Response to Post‐Publication Concerns About Clinical Research in Women's Health
Source: BJOG. 2025 Feb 26;132(7):892–901. doi: 10.1111/1471-0528.18100 (PMC12051221; doi:10.1111/1471-0528.18100)
Supplement: Supplementary file 3 — Data S1. Supporting Information. [file BJO-132-892-s002.docx]

**Supplementary Table 3A: Examples of Unresolved Cases**

| Author | Reference | Initial E-mail date | Response | Outcome |
| --- | --- | --- | --- | --- |
| Caliskan | AJOG 2002;187:1038-45.^(1)^ | 31-03-22 | AJOG never responded |  |
| Caliskan | Obstet Gynecol 2003;101:921-8 ^(2)^ | 31-03-22 | No further action | “no ample evidence of misconduct” |
| Guo | Reproductive Health 2022; 19:198 ^(3)^ | 04-04-24 | Confirmed receipt | Journal still investigating |
| Rezk | Pregn Hypertens 2016;6:291-94 ^(4)^ | 07-11-21 | Journal never responded | Journal still investigating |
| Selman | Fertil Steril 2010;94:1782-6 ^(5)^ | 04-01-24 | Confirmed receipt | Journal still investigating |
| Kumar | Int J Gyn Obstet 2005;89:7-13^(6)^ | 03-04-21 | None | Journal still investigating |
| Darwish | BMC Preg Childb. 2022;22:650. | 08-04-24 | Confirmed receipt | Journal still investigating |
| 1. Calişkan E, Meydanli MM, Dilbaz B, Aykan B, Sönmezer M, Haberal A. Is rectal misoprostol really effective in the treatment of third stage of  labor? A randomized controlled trial. *Am J Obstet Gynecol* 2002; 187: 1038-1045.  2. Caliskan E, Dilbaz B, Meydanli MM, Oztürk N, Narin MA, Haberal A. Oral misoprostol for the third stage of labor: a randomized controlled  trial. *Obstet Gynecol* 2003; 101: 921-928.  3. Guo H, Du T, Gao H, Xi Q, Wu L, Lyu Q, et al. The comparison of two different protocols ultra-long versus medroxyprogesterone acetate in  women with ovarian endometriosis: a prospective randomized controlled trial. *Reprod Health* 2022; 19: 198.  4. Rezk M, Ellakwa H, Gamal A, Emara M. Maternal and fetal morbidity following discontinuation of antihypertensive drugs in mild to moderate  chronic hypertension: A 4-year observational study. *Pregnancy Hypertens* 2016; 6: 291-294.  5. Selman H, Pacchiarotti A, El-Danasouri I. Ovarian stimulation protocols based on follicle-stimulating hormone glycosylation pattern: impact on  oocyte quality and clinical outcome. *Fertil Steril* 2010; 94: 1782-6.  6. Kumar A, Jain S, Singh NP, Singh T. Oral versus high dose parenteral iron supplementation in pregnancy. *Int J Gyn Obstet* 2005; 89: 7-13. | | | | |
|  | | | | |
