# Supplementary material for: Publishers' Response to Post‐Publication Concerns About Clinical Research in Women's Health
Source: BJOG. 2025 Feb 26;132(7):892–901. doi: 10.1111/1471-0528.18100 (PMC12051221; doi:10.1111/1471-0528.18100)
Supplement: Supplementary file 4 — Data S2. Supporting Information. [file BJO-132-892-s004.pptx]

## Slide 1
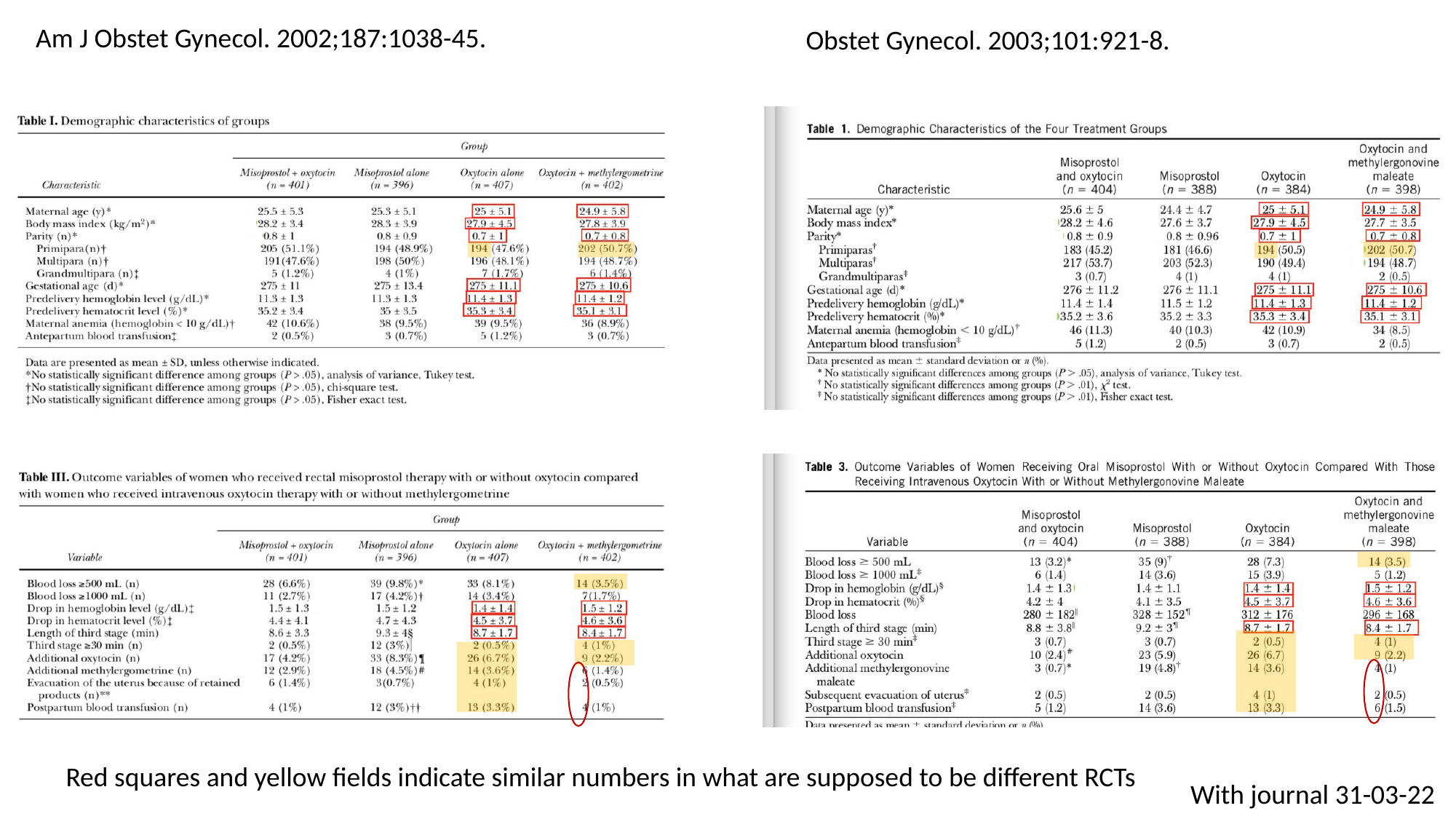

Am J Obstet Gynecol. 2002;187:1038-45.
Obstet Gynecol. 2003;101:921-8.
Red squares and yellow fields indicate similar numbers in what are supposed to be different RCTs
With journal 31-03-22

## Slide 2
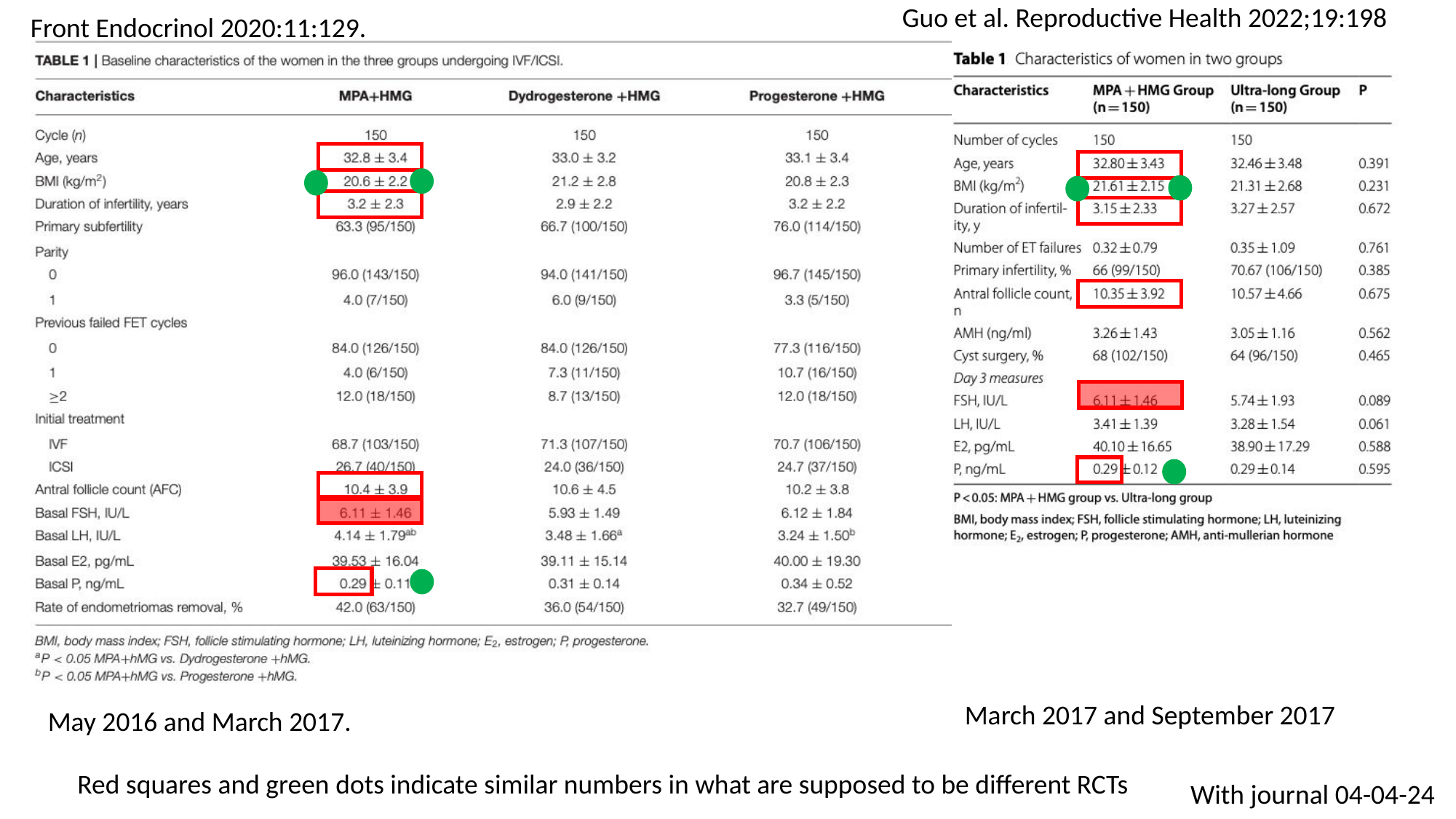

Guo et al. Reproductive Health 2022;19:198
Front Endocrinol 2020:11:129.
March 2017 and September 2017
May 2016 and March 2017.
Red squares and green dots indicate similar numbers in what are supposed to be different RCTs
With journal 04-04-24

## Slide 3
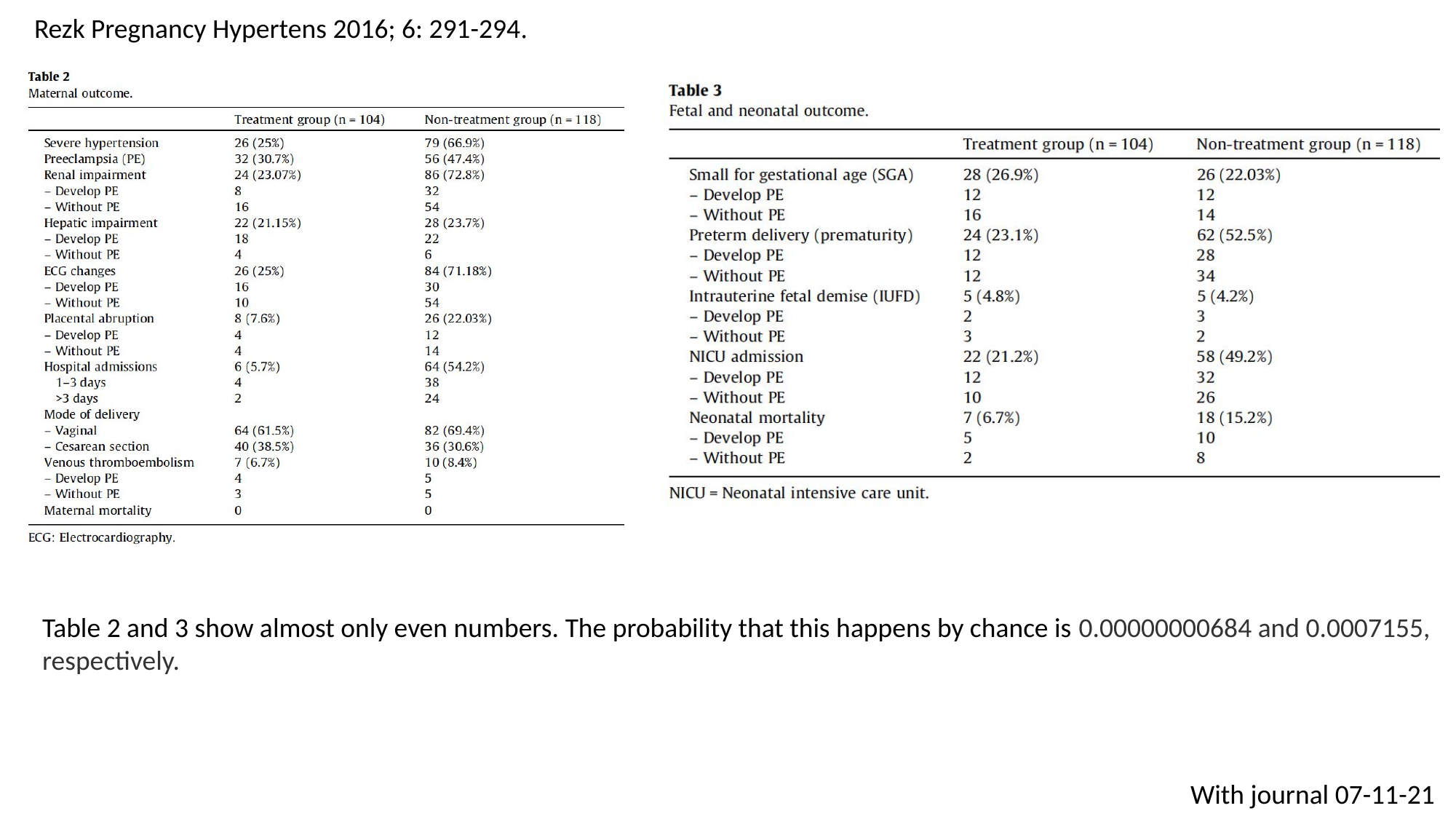

Rezk Pregnancy Hypertens 2016; 6: 291-294.
Table 2 and 3 show almost only even numbers. The probability that this happens by chance is 0.00000000684 and 0.0007155,
respectively.
With journal 07-11-21

## Slide 4
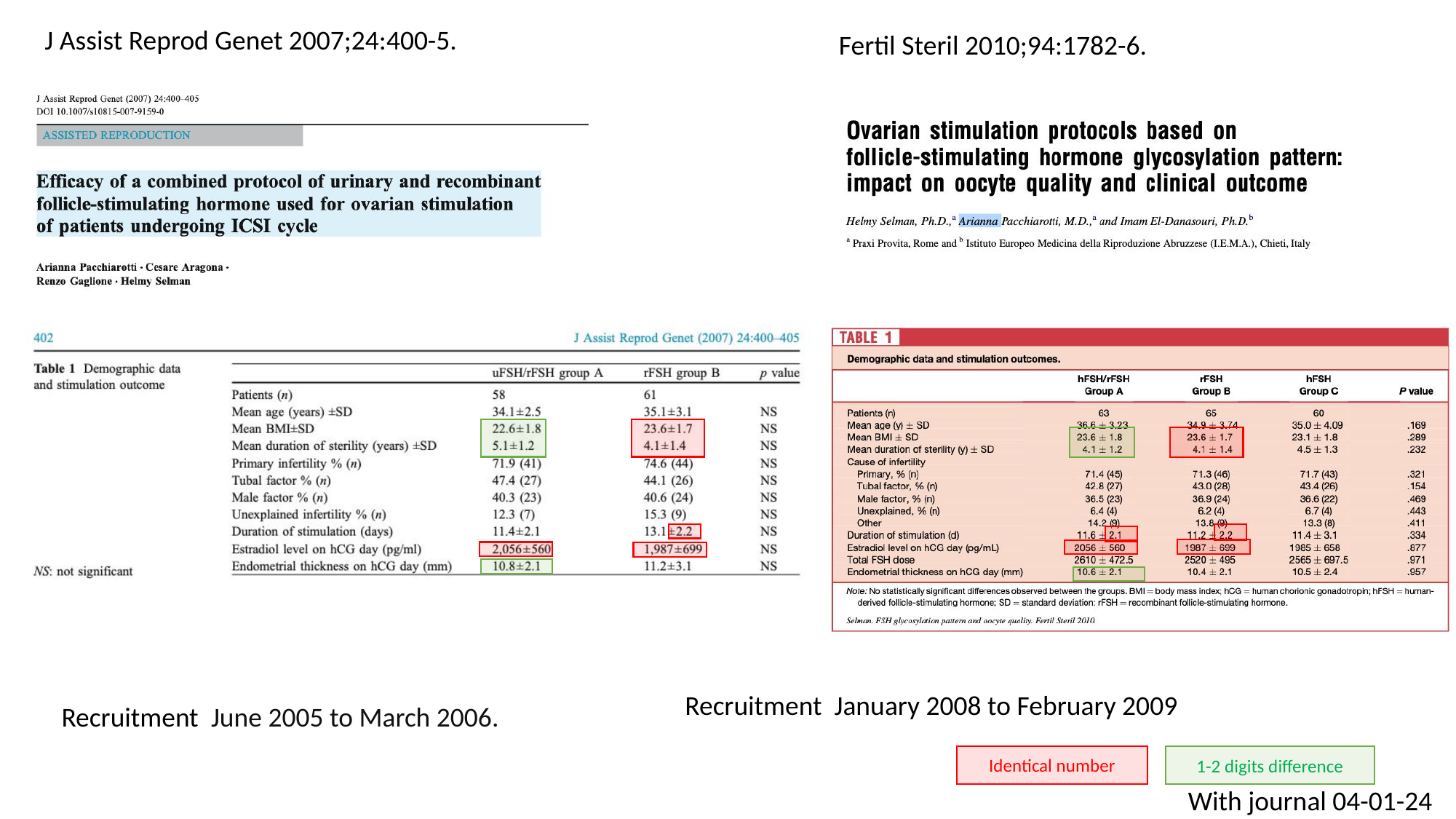

J Assist Reprod Genet 2007;24:400-5.
Fertil Steril 2010;94:1782-6.
Recruitment January 2008 to February 2009
Recruitment June 2005 to March 2006.
Identical number
1-2 digits difference
With journal 04-01-24

## Slide 5
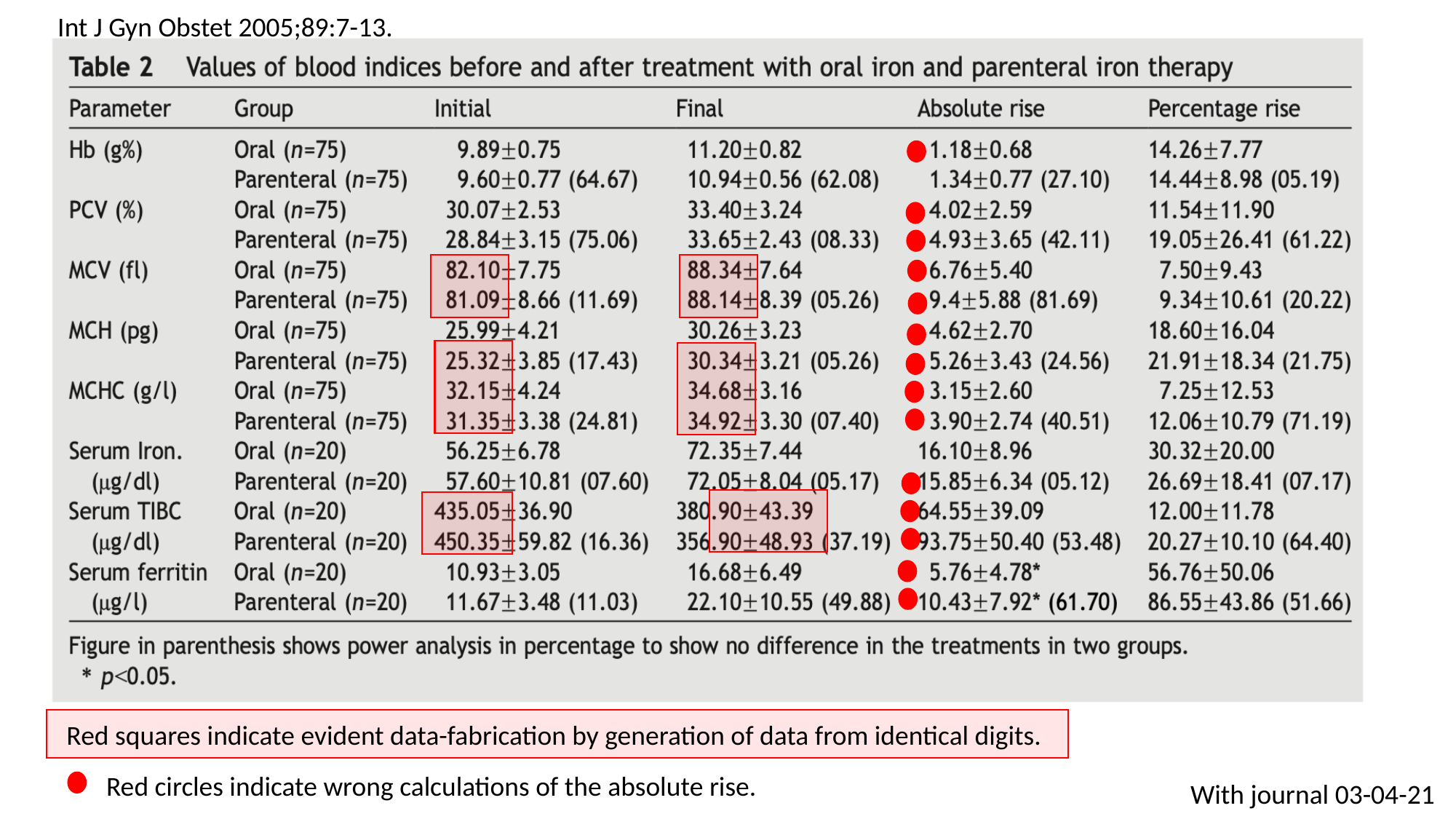

Int J Gyn Obstet 2005;89:7-13.
Red squares indicate evident data-fabrication by generation of data from identical digits.
Red circles indicate wrong calculations of the absolute rise.
With journal 03-04-21

## Slide 6
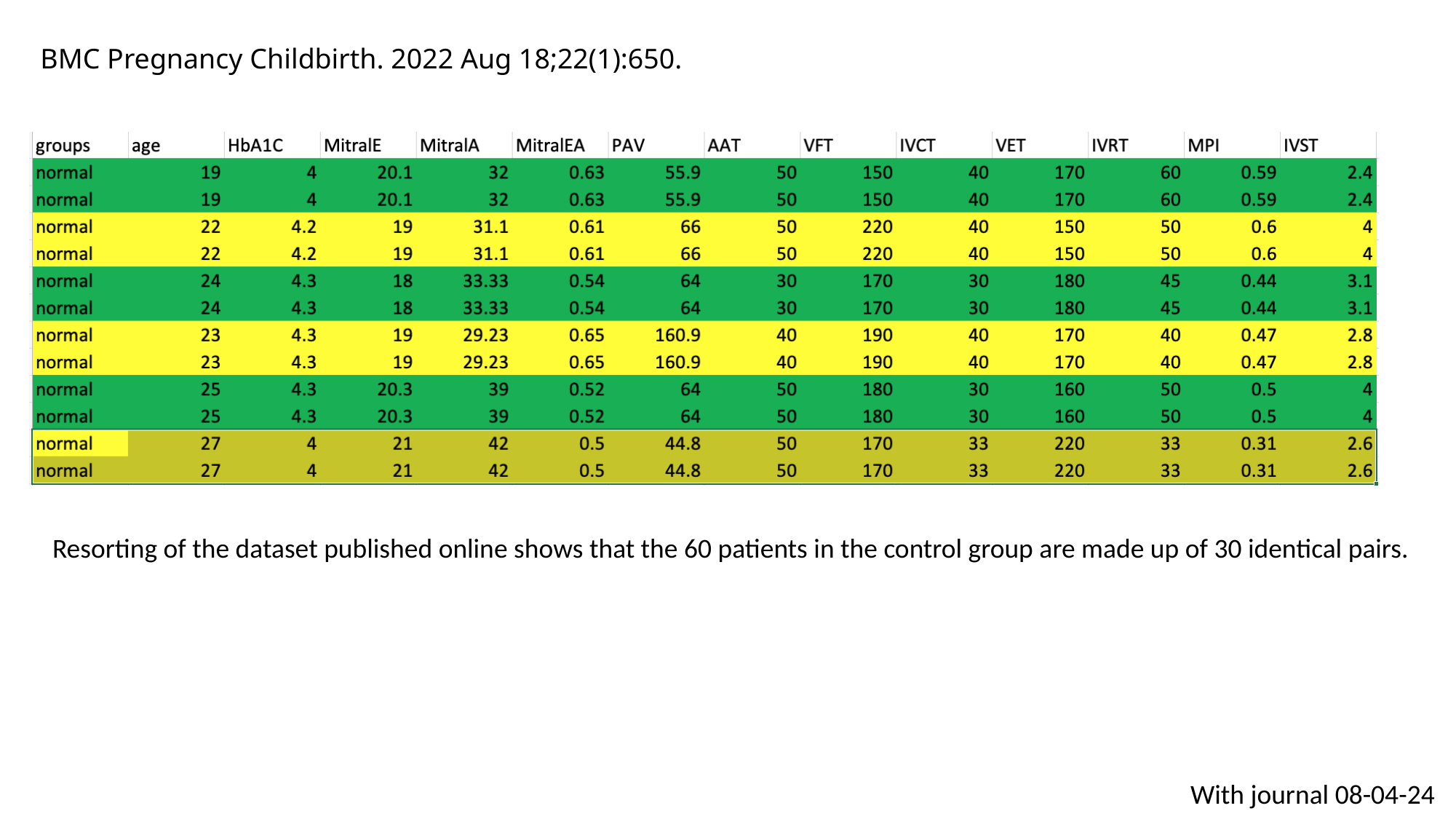

BMC Pregnancy Childbirth. 2022 Aug 18;22(1):650.
Resorting of the dataset published online shows that the 60 patients in the control group are made up of 30 identical pairs.
With journal 08-04-24
